# Lipidomics reveal the protective effects of a vegetable-derived isothiocyanate against retinal degeneration

**DOI:** 10.12688/f1000research.19598.5

**Published:** 2020-10-05

**Authors:** Faith A. Kwa, Nabeela K. Dulull, Ute Roessner, Daniel A. Dias, Thusitha W. Rupasinghe

**Affiliations:** 1 Discipline of Laboratory Medicine, School of Health and Biomedical Sciences, Royal Melbourne Institute of Technology University, Bundoora, Victoria, 3083, Australia; 2 Department of Health Sciences and Biostatistic, School of Health and Biomedical Sciences, Swinburne University of Technology, Victoria 3122, Australia; 3 Metabolomics Australia, School of BioSciences, The University of Melbourne, Parkville, Victoria, 3010, Australia

**Keywords:** Age-related macular degeneration, fatty acid, L-Sulforaphane, lipidomics, oxidative stress, retinal pigment epithelium

## Abstract

**Background:** Age-related macular degeneration (AMD) is a leading cause of blindness in the ageing population. Without effective treatment strategies that can prevent disease progression, there is an urgent need for novel therapeutic interventions to reduce the burden of vision loss and improve patients’ quality of life. Dysfunctional innate immune responses to oxidative stress observed in AMD can be caused by the formation of oxidised lipids, whilst polyunsaturated fatty acids have shown to increase the risk of AMD and disease progression in affected individuals. Previously, our laboratory has shown that the vegetable-derived isothiocyanate, L-sulforaphane (LSF), can protect human adult pigment epithelial cells from oxidative damage by upregulating gene expression of the oxidative stress enzyme Glutathione-S-Transferase µ1. This study aims to validate the protective effects of LSF on human retinal cells under oxidative stress conditions and to reveal the key players in fatty acid and lipid metabolism that may facilitate this protection.

**Methods:** The *in vitro* oxidative stress model of AMD was based on the exposure of an adult retinal pigment epithelium-19 cell line to 200µM hydrogen peroxide. Percentage cell proliferation following LSF treatment was measured using tetrazolium salt-based assays. Untargeted fatty acid profiling was performed by gas chromatography-mass spectrometry. Untargeted lipid profiling was performed by liquid chromatography-mass spectrometry.

**Results:** Under hydrogen peroxide-induced oxidative stress conditions, LSF treatment induced dose-dependent cell proliferation. The key fatty acids that were increased by LSF treatment of the retinal cells include oleic acid and eicosatrienoic acid. LSF treatment also increased levels of the lipid classes phosphatidylcholine, cholesteryl ester and oxo-phytodienoic acid but decreased levels of phosphatidylethanolamine lipids.

**Conclusions:** We propose that retinal cells at risk of oxidative damage and apoptosis can be pre-conditioned with LSF to regulate levels of selected fatty acids and lipids known to be implicated in the pathogenesis and progression of AMD.

## Introduction

Age-related macular degeneration (AMD) is a major cause of blindness worldwide, especially targeting the ageing population. AMD is categorised into three main stages, namely early, intermediate and late AMD. The early stage is marked by the thickening and inflammation of the Bruch’s membrane, as a result of the accumulation of fatty proteins known as drusen ^1^. The intermediate stage proceeds with an increase in size of these drusen particles, resulting in pressure atrophy on the retinal pigment epithelium (RPE) and thinning of the macula (dry AMD), which results in the deterioration of central vision. In one of the late stages of AMD (wet AMD), the atrophic retinal tissue becomes replaced with granulation tissue consisting of abnormal leaky blood vessels ^1^. The blood and fluid leak from these blood vessels into the retina; thus, prolonging the chronic inflammatory response and triggering further oxidative damage. Many factors contribute to AMD. One dominant factor is the increasing age of the retina, where the RPE becomes damaged due to a progressively impaired DNA repair system that fails to repair oxidative damage from prolonged exposure to visible light, ultraviolet A and reactive oxygen species (ROS) over time ^2^. Cigarette smoking is another factor that contributes to the production of ROS and oxidative damage on the RPE layer. Many studies have shown a link between excessive cigarette smoking and AMD ^3, 4^. For the retina to maintain its normal physiological functions, a well-balanced diet is also necessary. Poor nutrition in the elderly influences the progression of AMD. Studies by Rochtchina *et al*. (2007) and Gopinath *et al*. (2013) showed that a deficiency of Vitamin B12 is linked to an increased risk of AMD ^5, 6^. Despite recent evaluations of stem cell–derived therapeutic approaches in Phase I clinical trials, such novel methods require long-term use of immunosuppressive drugs, which may lead to other medical implications ^7^. Conventional therapies include FDA-approved anti-angiogenic agents, thermal laser photocoagulation or intravitreal injection of medications aim to limit neovascularisation ^8^. However, these treatments do not cure AMD but mainly reduce patients’ symptoms and usually target the late stages of the disease where abnormal neo-vacularisation is observed. Therefore, further studies must be carried out to find an effective preventative measure, especially in targeting early stages of the disease before the onset of vision loss.

In oxidative stress conditions including those observed in the retina of patients with AMD, redox enzymes coded by genes such as *glutathione-S-transferase µ1 (GSTM1)* are important in preventing ROS accumulation in the retina but these genes are often expressed in low levels in AMD patients ^2^ In later stages of AMD (i.e. ‘exudative’ AMD), genes such as *Vascular Endothelial Growth Factor A (VEGFA)* play a vital role in its development ^8, 9^. Previously, imbalanced levels of fatty acids responsible for the abnormal function of the retina were associated with AMD progression. There are five major fatty acids in the human retina, namely, docosahexaenoic acid (DHA), arachidonic acid (ACA), stearic acid, oleic acid and palmitic acid. Both DHA and ACA are classified as long chain polyunsaturated fatty acids (LC-PUFAs) ^10^. It was reported that a deficiency in docosahexaenoic acid and arachidonic acid interfere in neurological and visual signalling pathways, and intake of these LC-PUFAs increased the risk of AMD ^10, 11^. In addition, other studies found that ROS produced during oxidative stress can damage the essential PUFAs in the retina and generate toxic lipid peroxidation end products (i.e. reactive aldehydes 4-hydroxynoneal and 4-hydroxyhexenal); thus, exacerbating the chronic-inflammatory damage in the retina. These accumulated aldehydes can in turn, inhibit redox enzyme reactions, DNA and RNA synthesis and biosynthesis of proteins ^12^. PUFAs are an important substrate for redox enzymes such as glutathione S transferases ( *GST*s) during oxidative stress-mediated lipid peroxidation and healthy fatty acid (FA) levels are crucial for the efficient removal of ROS from the retina ^13, 14^. Dysfunctional innate immune responses to oxidative stress observed in AMD are also reported to be attributed to the formation of oxidized lipids ^15^. Therefore, lipid and fatty acid pathways remain vital in maintaining a healthy environment in the retina. Furthermore, patients with AMD were reported to have low levels of other metabolites, such as glucose, lactate, glutamine and albumin, suggesting the possible role of a dysregulated metabolome in this disease ^16, 17^. As such, the pathogenesis of AMD is likely to involve the abnormal expression of *VEGFA*, *GSTM1* and imbalanced levels of selective metabolites, such as fatty acids. This prompts the investigation of new and potential therapeutic agents that can alleviate the aberrant gene expression via chromatin remodelling processes and restore normal levels of metabolites in the retina.

Here, we propose the use of L-Sulforaphane (LSF), a naturally occurring isothiocyanate found in many cruciferous vegetables like broccoli in the treatment of AMD ^18^. LSF has been shown to have epigenetic properties in solid tumours by enhancing the acetylation of histones, resulting in an ‘opened’ chromatin state, which triggers the transcription of genes involved in cell death and restores the apoptotic potential of cancer cells ^19, 20^. These anti-carcinogenic effects have also been associated with downregulation of the pro-inflammatory marker, hypoxia inducing factor 1-α, and VEGF while increasing redox enzyme activities ^21, 22^. Such antioxidant properties could be useful for the treatment of the AMD. Whilst it has the potential to induce cell death in malignant cancer cells, it can protect retinal tissue from photoreceptor degeneration under oxidative stress conditions ^23^. This protection is mediated via the induction of phase II detoxification enzyme *NAD(P)H:quinoxidoreductase* and transcriptional activation of antioxidant response element; thus elevating glutathione levels in the retinal cells ^23^. Hence, the action of LSF is unique and seems to be disease specific. This characteristic enables LSF to be considered a potential drug candidate in targeted therapy.

In 2018, our laboratory reported the ability of LSF at micro molar concentrations (3µM and 5µM) to protect human retinal pigment epithelium from cell death and promoted the regeneration of these cells under oxidative stress conditions ^24^. This preliminary study involving gas chromatography mass spectrometry (GC-MS) analytical methods revealed that LSF treatment induced changes in the levels of FAs, such as nonanoic acid and 9,12,15- (Z-Z-Z)-Octodectrienoic acid, and upregulated the levels of *GSTM1* gene expression ^24^. However, many of these significant changes were observed with the 5µM LSF treatment. These findings have warranted the current study to further examine lipids and fatty acids that may regulate the antioxidant effects of LSF. In the current study, dose response data using LSF concentrations of 3–30µM validate the previously reported protective and regenerative properties of this compound against oxidative stress, where a dose-dependent increase in cell proliferation is observed and then plateaus at a concentration higher than 20µM. For the first time, we report the use of a lipidomic approach using liquid chromatography with triple-quadrupole mass spectrometry (LC- *QqQ*-MS) to analyse human retinal pigment epithelial (ARPE-19) cells pre-treated with 5 and 20µM LSF under oxidative stress conditions. The total pool of FAs affected by the treatment will be confirmed by gas chromatography-mass spectrometry (GC-MS) and used to putatively identify lipid classes. We hypothesize that LSF can increase the levels of lipids containing unsaturated FAs while decreasing levels of lipids with saturated FAs for the protection of ARPE-19 cells against oxidative damage.

## Methods

### Cell culture

The Adult Retinal Pigment Epithelium-19 (ARPE-19) cell line was purchased from the American Type Cell Collection (USA). The cells were cultured in complete Dulbecco’s Modified Eagle Medium (DMEM/F12) containing 200mM L-glutamine and 15mM HEPES (Life Technologies, USA). The culture media was further supplemented with 10% foetal calf serum (FCS; Sigma Aldrich, USA) and 1% penicillin-streptomycin 10,000 U/ml (Life Technologies, USA). The ARPE-19 cells were sustained at 37 **°**C in an atmosphere of 95% air and 5% CO _2_, and phenotypic characteristics of these cells were validated in our previous publication ^24^.

### Cell treatment prior to analysis

The ARPE-19 cells were starved in a serum-deprived DMEM/F12 media containing 1% FCS and 1% penicillin-streptomycin for 24 hours. For the CellTiter 96 AQueous One Solution Cell Proliferation (MTS) Assay, the cells were exposed to 0.025% dimethyl sulfoxide (DMSO; Sigma Aldrich, USA) as the drug vehicle control or 3µM LSF, 5µM LSF, 10µM LSF, 20µM LSF or 30µM LSF for 24 hours. For the GC-MS/LC-MS analysis, the cells were exposed to 0.025% DMSO, 5µM LSF or 20µM LSF for 24 hours. The negative control for all analyses was untreated cells that were incubated in serum-deprived DMEM/F12 media. The term “untreated cells” refers to cells not treated with LSF regardless of the oxidative stress stimulus being present or not. After 24 hours incubation, the treatments were discarded from all the wells and the cells were incubated with 200µM hydrogen peroxide (H _2_O _2_; Sigma Aldrich, USA) as an oxidative stress stimulus for two hours. Untreated cells or LSF-treated cells incubated in Hanks Balanced Salt Solution (HBSS; Sigma Aldrich, USA) for two hours were used as the negative control for oxidative stress. Subsequently, the H _2_O _2 _or HBSS was removed and the cells were allowed to recover for 24 hours in serum-deprived DMEM/F12 media before either the MTS assay or the GC-MS/LC-MS analysis were carried out ^24^.

### CellTiter 96 AQueous One Solution Cell Proliferation Assay (MTS)

The ARPE-19 cells were trypsinised using 0.25% trypsin EDTA (Life Technologies, USA) and centrifuged at 200 g for three minutes, before being seeded at a density of 10 ^5^ cells/mL in 100µL of complete cell culture media in 96-well flat-bottom plates and treated with the agents described above. Each well contained 10,000 cells. To assess the effects of LSF treatment in the presence or absence of oxidative stress on cell proliferation, the MTS assay (catalogue number G3580; Promega, USA) was carried out according to the manufacturer’s protocol and as previously described ^24^. A volume of 20µL MTS reagent was added to the cells in each well and plate was incubated for four hours at 37°C and in an atmosphere of 95% air and 5% CO _2_. Absorbance readings (at 490nm) of drug-treated cells were normalised to the untreated control. As per the manufacturer’s protocol, % cell proliferation = (Absorbance _drug treatment_ – Absorbance _blank_) / (Absorbance _untreated_ – Absorbance _blank_) × 100%. The percentage of cell proliferation was calculated as the mean of results from three independent experiments with three technical replicates per experiment.

### Harvesting of treated ARPE-19 Cells for GC-MS and LC-MS analysis

The ARPE-19 cells were seeded at a density of 1.5×10 ^6^ per well in 6-well plates and conditioned as indicated above. The cells were removed using 0.25% trypsin EDTA, followed by centrifugation at 200 g for three minutes. The cell pellets were resuspended in ice-cold 1X phosphate-buffered saline (pH 7.4) and the number of live cells were counted by trypan blue exclusion before being transferred to microcentrifuge tubes. These tubes were centrifuged twice at 200 g for three minutes and after each spin, the pellets were resuspended in ice-cold PBS (washing step). The tubes were spun a third time, the supernatant was removed to remove any remaining dead cells and cell debris. The pellets were frozen at -80°C to be used for the extraction. Four replicates of each control and treated samples were performed.

### Extraction of fatty acids and lipids from treated ARPE-19 Cells for GC-MS and LC-MS analysis

Upon cell harvesting, each cell pellet was washed with 200 μL of water by vigorous vortexing for 19 seconds. A volume of 250 μL of methanol and 0.01% butylated hydroxytoluene (v/v) mixture was added to the cell pellets. The samples were then frozen for five minutes in liquid nitrogen, followed by sonication for another five minutes at room temperature at 100 rpm. The freeze-sonication steps were then repeated three times to lyse the cell pellets. The lysed cells were then vortexed vigorously for one minute. A volume of 500 μL of chloroform was added to the lysate and was mixed for 30 minutes at room temperature using a shaker. Next, the samples were centrifuged at 14,100g, 5°C for 15 minutes. The supernatant from each sample was transferred into respective clean 1.5 mL Eppendorf tubes (Tube A). A mixture containing 500 μL of chloroform:methanol (2:1) (v/v) was added to the cell pellets as the second extraction step. The samples were vortexed for 30 seconds and shaken for 15 minutes at room temperature before centrifugation at 16,100g, 0°C for 15 minutes. The supernatant from the second extraction was then combined into the supernatant in the respective Tube As. The combined supernatant for each sample was dried down under a stream of nitrogen. Each dried lipid extract was resuspended in 200 μL of butanol:methanol (1:1) (v/v) with 10 mM ammonium formate for LC-MS analysis ^25^. Additionally, a 30 μL aliquot was transferred into a glass insert and dried *in vacuo* for subsequent fatty acid methyl ester (FAME) analysis on the GC-MS. All samples were stored in the dark in bags containing silica beads prior to GC-MS and LC-MS analysis.

### FAME analysis using GC-MS

The dried ARPE-19 cell extracts were resuspended in chloroform:methanol (2:1 v/v) (25µL) containing 60 μM of the internal standard ( ^13^C-labelled myristic acid), followed by the addition of the derivatizing agent (5µL) (catalogue number 11370591, Meth-Prep II™, Grace Davison Discovery, Deerfield, IL, US,). Each sample was subsequently incubated at 37°C for 30 min, then held for 10 min at room temperature. Then, 1 μL of the derivatised ARPE-19 cell extract was injected onto the GC-MS system consisting of a Gerstel 2.5.2 autosampler (catalogue number G7368A), a 7890A Agilent gas chromatograph (catalogue number G3440B), and a 5975C Agilent quadrupole MS (catalogue number G7042A) (Agilent Technologies, Santa Clara, US). The FAME analysis which measures the level of fatty acids was carried out using a 30 m column with a 0.25 μm film thickness, 0.25 mm inner diameter and a 10 m guard column (catalogue number CP8944, Agilent J&W Scientific VF-5MS GC Column). The following parameters were set for GC-MS FAME analysis: injection port temperature (250°C), MS transfer line (280°C), ion source temperature (230°C) and quadrupole (150°C). The carrier gas used for the analyses was helium (UHP 5.0) at a flow rate of 1.0 mL/min. For the FAME analysis, the temperature program used was: start at injection (50°C), hold for one min followed by a 15°C.min ^-1^ oven temperature ramp to 230°C, hold for three min followed by a 10°C.min ^-1^ oven temperature ramp to 325°C and a final three min heating at 325°C. Mass spectra were recorded at two scans/s with a 50–600 *m/z* scanning range ^26^. Detected fatty acids were annotated as: first two digits as sum of carbon atoms in the fatty acid chains followed by a single digit that indicates the sum of double bonds in the fatty acid chains.

### Lipid analysis using LC-MS

Lipid analysis using LC-MS was carried out as published previously ^25^. Briefly, to separate the lipids, 5 µL aliquots per sample were injected onto a 50 mm × 2.1 mm × 2.7 µm Ascentis Express RP Amide column (catalogue number 53911-U, Supelco, Sigma, St Louis, USA) at 35°C using an Agilent LC 1200 (Mulgrave, Australia).

Lipid detection was carried out using Agilent 6410 triple quad (catalogue number, 6410, Mulgrave, Australia) in electrospray ionisation (ESI) mode. Lipid species were identified based on the lipid class using precursor ion and Neutral loss scanning techniques as discussed previously ^25^. Diacylglycerol and triacylglycerol species were identified based on the neutral loss of fatty acyl moiety.

Identified lipid species were quantified via multiple reaction monitoring (MRM) with a 20 ms dwell time for the simultaneous measurements of ~20 to 50 compounds and the chromatographic peak width of 30 sec to 45 secs. A minimum of 12 to 16 data points was collected across the peak. Optimised parameters for capillary, fragmented, and collision voltages were 4000 V, 140 – 380, and 15–60 V, respectively. The collision gas used was nitrogen at 7 Lmin ^-1^. 

The lipidomic data was generated using reverse phase peak area response of each lipid series rather than absolute concentrations. Furthermore, to compare the lipid levels between untreated and treatment groups, the data has been normalised to the number of cells per sample, and the median of the reverse phase peak area response was log _2_ transformed. An auto-scale has also been applied. Detected lipid species were annotated as lipid class (i.e. sum of carbon atoms in the fatty acid chains: sum of double bonds in the fatty acid chains).

### Statistical and data analyses

Significant changes in cell proliferation and levels of total fatty acids or lipid species were validated by one-way analysis of variance and the post-hoc Bonferroni, and paired t-test to determine any significant differences between the LSF-treated groups and untreated or vehicle controls. The Bonferroni method which is a standard p-value correction tool provided in MetaboAnalyst provides the strongest control of the false positives and therefore confers a high confidence in the selected metabolic features particularly for the purposes of novel untargeted studies like the current study. The GC-MS and LCMS ESI-MRM data was processed using Agilent Mass Hunter Quantitative Analysis soft-ware (Version 6) (Mulgrave, Australia). The heatmaps and boxplots were generated using the open-source tool, ‘MetaboAnalyst 3.0’ ( http://www.metaboanalyst.ca) (USA) ^27^. When using the MetaboAnalyst software to perform statistical analysis, if the real peaks detected are close to background noise or are not detected in one group, MetaboAnalyst will provide the significant differences with the standard deviation of the replicates within the group taken into consideration. Therefore, any falsely identified peaks reporting as significant peaks will be eliminated.

## Results

### Effects of hydrogen peroxide on the proliferation of ARPE-19 cells

**Figure 1.**
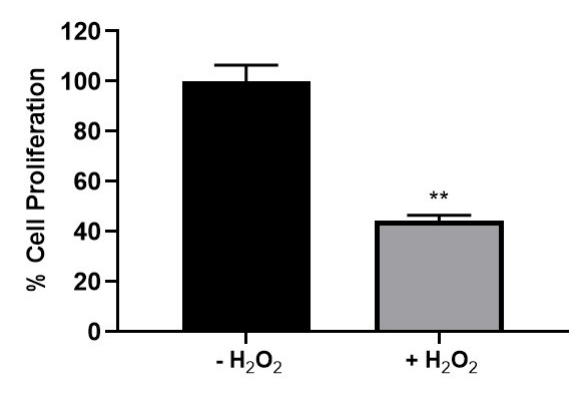
Net effect of H _2_O _2_ on ARPE-19 cell proliferation. The cells were treated with 200 µM hydrogen peroxide (H _2_O _2_) for two hours (+H _2_O _2_) or were left untreated (-H _2_O _2_). Cells in both groups were not treated with L-sulforaphane. The mean absorbance values for +H _2_O _2_ group are presented as a percentage of that of the -H _2_O _2_ untreated control group (n= 3; **p < 0.01)

The effects of H _2_O _2_ on the percentage cell proliferation of ARPE-19 cells were determined by the MTS assay. Figure 1 shows that the exposure of these cells to 200 µM H _2_O _2_ for two hours led to a 55.8% reduction in cell proliferation. These results show a consistent trend with those published in our previous article ^24^.

### Effects of LSF on the proliferation of ARPE-19 cells in the presence or absence of oxidative stress

**Figure 2.**
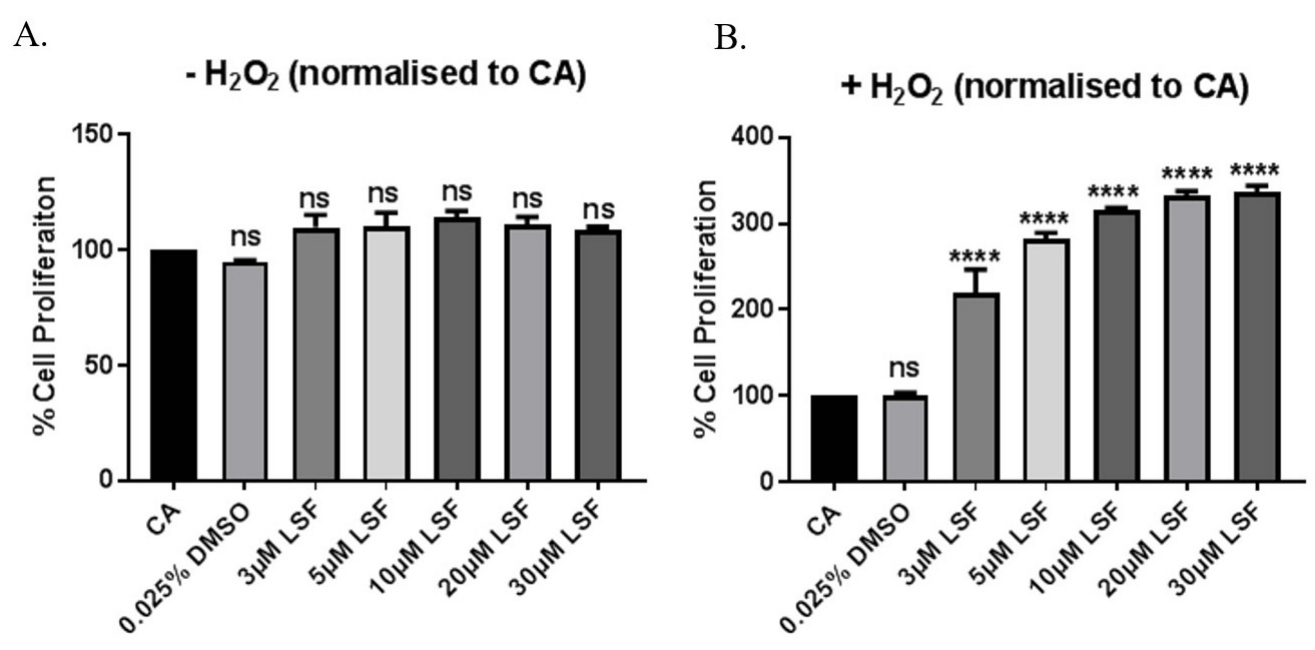
Effects of LSF on ARPE-19 cell proliferation in ( **A**) without or ( **B**) with H _2_O _2_. The proliferative effects of vehicle control (0.025% DMSO) and 3 µM - 30 µM LSF on cells were determined. The cells were treated with 0.025% DMSO or LSF for 24 hours prior to exposure with 200 µM H _2_O _2_ for two hours. The mean absorbance values for each treatment group are presented as a percentage of that of their respective untreated controls (CA) in the absence or presence of H _2_O _2_. (n= 3; not significant (ns): *p* > 0.05, and **** *p* < 0.0001). [CA, cells alone; DMSO, dimethyl sulfoxide; H _2_O _2_, hydrogen peroxide; LSF, L-Sulforaphane].

The drug vehicle control (0.025% DMSO) did not affect the percentage of proliferation regardless of exposure to oxidative stress stimulus, H _2_O _2_ ( Figure 2; all *p* values > 0.05) ^28^. In the absence of H _2_O _2_, 3 µM - 30 µM LSF treatments did not have a significant impact on cell proliferation ( Figure 2A; all *p* values > 0.05). In contrast, a dose-dependent increase in the proliferation of LSF-treated cells was observed at doses of 3µM to 20 µM under H _2_O _2_ conditions ( Figure 2B; all *p*-values < 0.0001). Increasing the dose to 30µM LSF did not induce any further increase in cell proliferation ( Figure 2B: vs 20µM, *p* value > 0.9999). These results validate the ability of LSF to protect ARPE-19 cells against oxidative stress by stimulating the regeneration of these cells. Henceforth, GC-MS and LC-MS analyses were performed on cells treated with the lowest and highest doses of LSF that resulted in significant increases in cell proliferation (i.e. *p* < 0.0001) compared to the untreated cells as the control group. Since there were no significant differences in cell proliferation between 20 µM and 30 µM LSF treatment groups, 30 µM LSF was not included in the GC-MS and LC-MS analyses.

### Effects of LSF on the total fatty acid and lipidome in ARPE-19 cells in the absence or presence of oxidative stress

**Figure 3.**
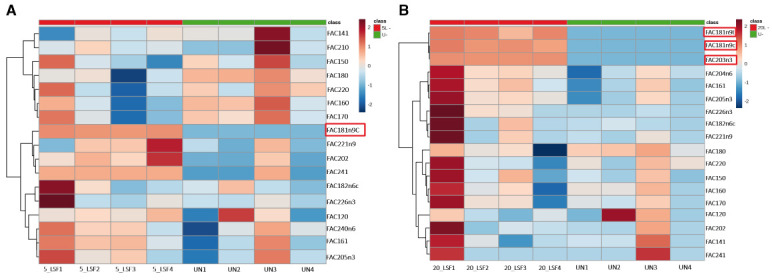
Total fatty acid levels in ( **A**) 5µM or ( **B**) 20µM LSF-treated ARPE-19 cells without H _2_O _2_. Four replicate samples for each treatment group were compared with that of the untreated controls without H _2_O _2_. Data in red and blue indicate an increase and decrease in FA levels, respectively. Total fatty acids (FACs) highlighted in red boxes show consistent changes in levels between the replicates within the treatment groups and those within the untreated control group. [5_LSF: 5µM LSF; 20_LSF: 20µM LSF. FA, fatty acid; FAC, total fatty acid; FAC 18:1 n9c, *cis*-oleic acid; FAC 18:1 n9t, *trans*-oleic acid; FAC 20:3, eicosatrienoic acid; H _2_O _2_, hydrogen peroxide; LSF, L-Sulforaphane; UN, untreated].

**Figure 4.**
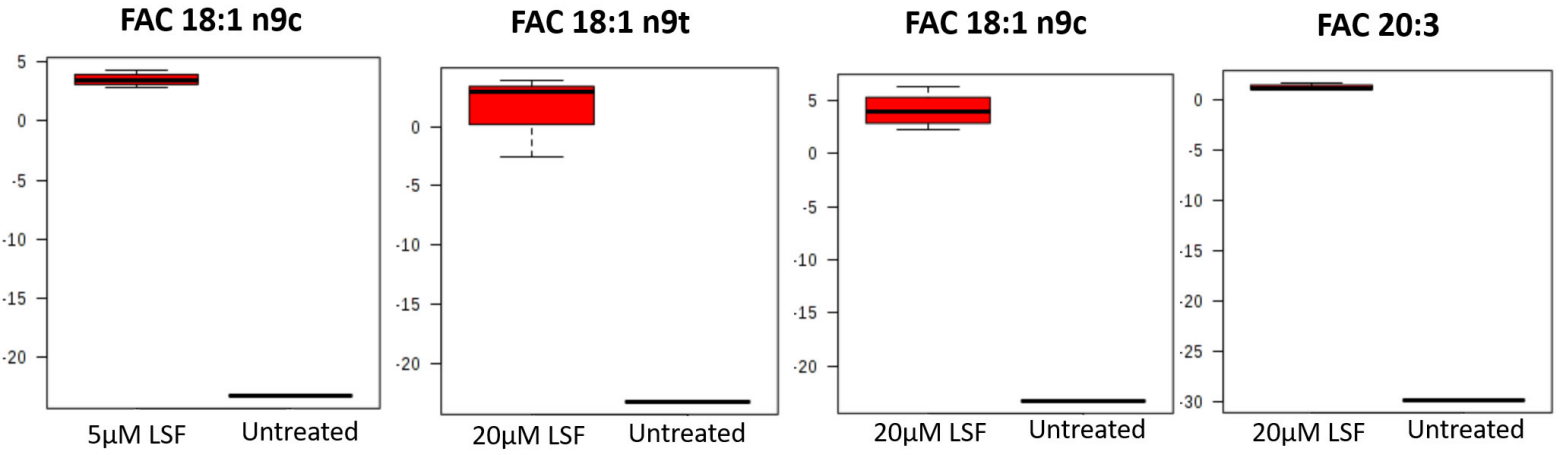
Changes in total fatty acid (FAC) levels in LSF-treated ARPE-19 cells without H _2_O _2_ compared to the untreated control without H _2_O _2_. Four replicate samples treated with either 5µM or 20µM LSF were compared with that of the untreated controls. Sample variation is depicted by the error bars. The y-axis values are automatically generated as arbitrary units by MetaboAnalyst software. [FAC, total fatty acid; FAC 18:1 n9c, *cis*-oleic acid; FAC 18:1 n9t, *trans*-oleic acid; FAC 20:3, eicosatrienoic acid; H _2_O _2_, hydrogen peroxide; LSF, L-Sulforaphane].

In the absence of oxidative stress, 5µM LSF treatment resulted in higher levels of the fatty acid, *cis*-oleic acid (18:1) while 20µM LSF treatment led to increased levels of *trans*-oleic acid, *cis*-oleic acid and eicosatrienoic acid (ETA) (20:3) in comparison to the untreated control ( Figure 3 and Figure 4). It is noteworthy that the levels of fatty acids in the untreated control fell below the level of detection and thus the missing values for the control were imputed, a common practice in metabolomics data analysis. In the presence of oxidative stress, there were no consistent differences in the fatty acid levels between the replicate samples within the 5μM LSF or 20μM LSF treatment groups and those within the untreated control group (see *Underlying data*) ^28^.

**Figure 5.**
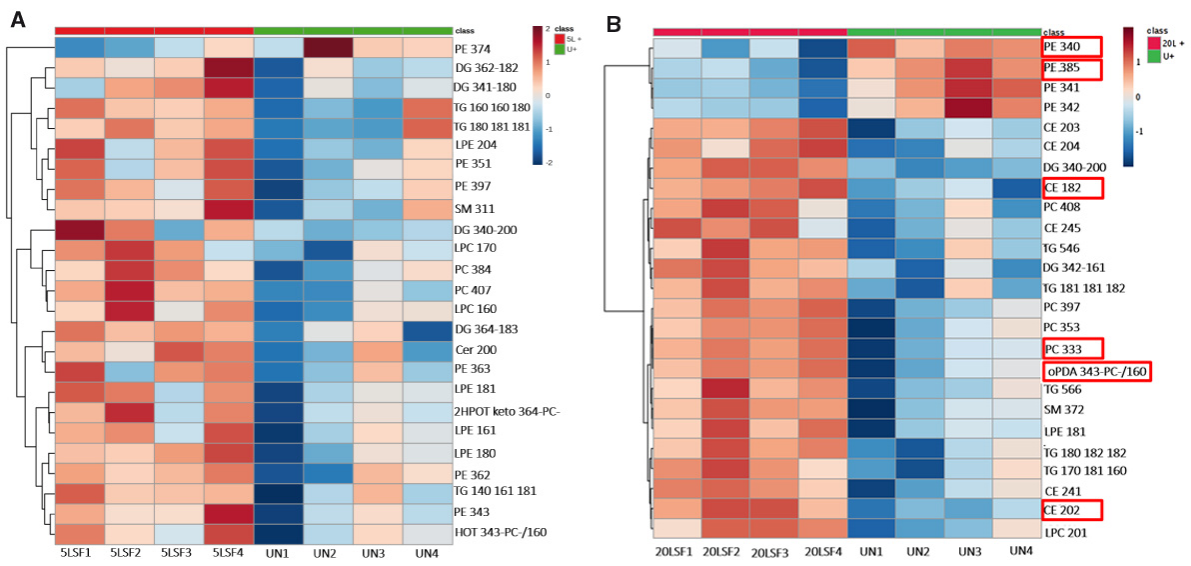
Lipid levels in ( **A**) 5µM LSF or ( **B**) 20µM LSF-treated ARPE-19 cells exposed to H _2_O _2_. Four replicate samples across the treatment groups were compared with that of the untreated [UN] controls exposed to H _2_O _2_. Data in red and blue indicate an increase and decrease in lipid levels, respectively. Lipids highlighted in red boxes show statistically significant changes in levels between treated groups and untreated controls ( *p* values < 0.05). [CE, cholesteryl ester; oPDA, oxo-phytodienoic acid; PC, phosphatidylcholine; PE, phosphatidylethanolamine; 5LSF, 5µM LSF; 20LSF, 20µM LSF; H _2_O _2_, hydrogen peroxide; LSF, L-Sulforaphane].

**Figure 6.**
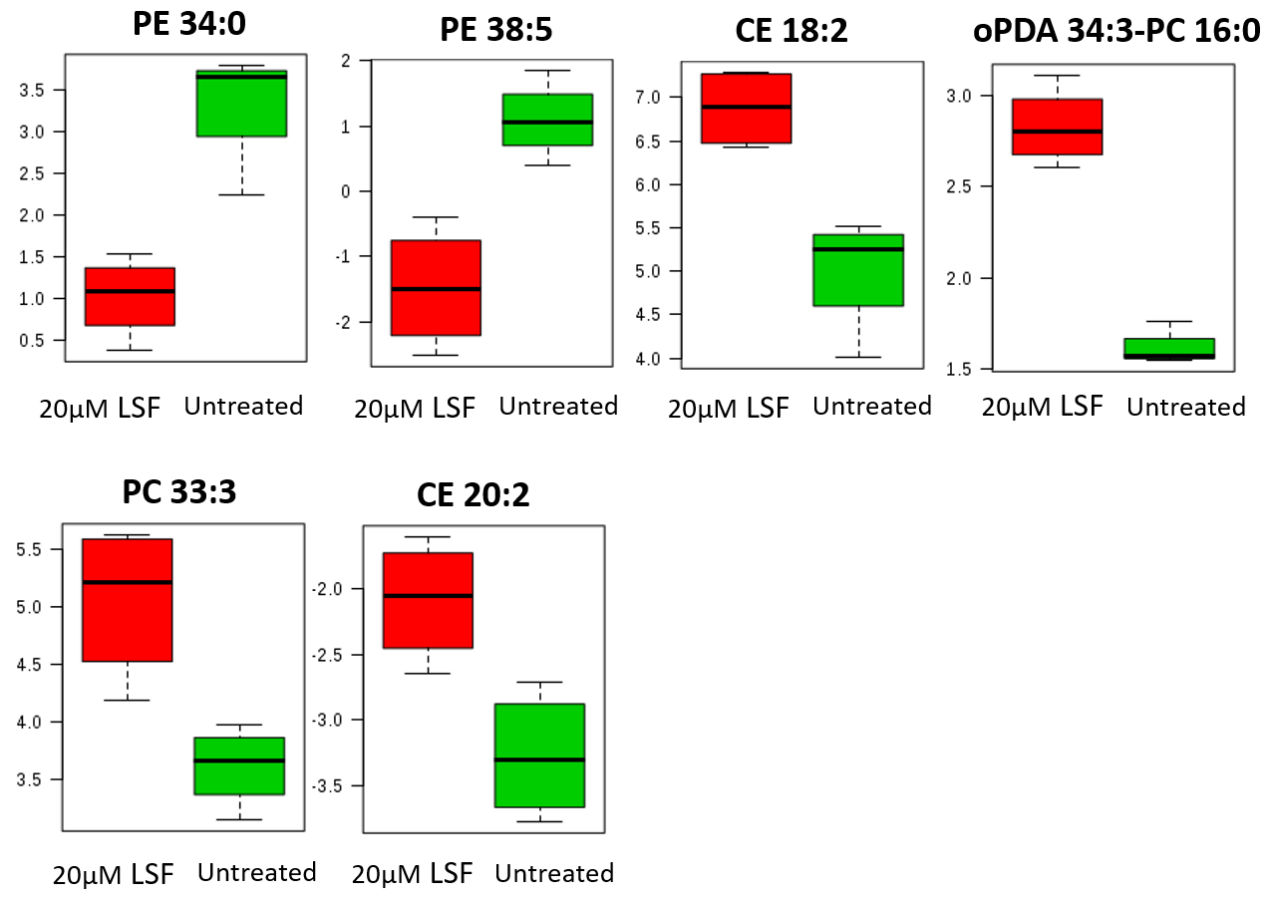
Significant changes in lipid levels in 20µM LSF-treated ARPE-19 cells exposed to H _2_O _2_. Four replicate samples treated with 20µM LSF were compared with that of the untreated controls. Both LSF-treated and untreated groups were exposed to H _2_O _2_. Sample variation is depicted by error bars. The y-axis values are automatically generated as arbitrary units by the Agilent Mass Hunter Quantitative Analysis software. [CE, cholesteryl ester; oPDA, oxo-phytodienoic acid; PC, phosphatidylcholine; PE, phosphatidylethanolamine; H _2_O _2_, hydrogen peroxide; LSF, L-Sulforaphane].

In the absence of oxidative stress, no changes in lipid levels between LSF-treated cells and the untreated control were reported (all *p* values > 0.05; see *Underlying data*) ^28^. In the presence of oxidative stress, treatment with 5μM LSF did not result in any statistically significant changes in lipid levels (all p values > 0.05; Figure 5A). However, significant changes were observed in the 20μM LSF treatment groups in the presence of oxidative stress ( Figure 5B). This study showed that LSF treatment increased levels of phosphatidylcholine (PC 33:3; p value = 0.0021409) by 2.133-fold and cholesteryl ester (CE 18:2 and CE 20:2; p values = 0.00344 and 0.0028703, respectively) lipids containing unsaturated FAs by 5.619-fold and 8.155-fold respectively, and oxo-phytodienoic acid (oPDA 34:3-PC 16:0; p value = 0.00062092) by 3.040-fold. However, LSF treatment decreased levels of phosphatidylethanolamine (PE 34:0; p value = 0.0018096) consisting of saturated FAs by 0.359-fold ( Figure 5B and Figure 6) was also observed. Other PE lipids containing unsaturated FAs (PE 38:5; p value = 0.00025506) were also decreased by 0.427-fold. The p values reported above derived from the paired t-test analysis. A one-way ANOVA with post-hoc analysis was also performed to determine any significant difference between the various six groups (untreated, 5µM LSF, 20µM LSF with and without H _2_O _2_. The p values indicate that differences in the levels of the 6 lipids were observed across the six groups (PE 34:0 p value = 0.0007042; PE 38:5 p value = 0.0000012584; CE 18:2 p value = 0.00083453; oPDA 34:3 p value = 0.0000085797; PC 33.3 p value = 0.0016225; and CE 20:2 p value = 0.0011231).

## Discussion

Since 2019, the World Health Organisation has classified AMD as one of the top 10 priority eye diseases and leading cause of blindness in the ageing population ^29^. Therefore, without a current cure, there is an urgent need for better prevention, treatment and management strategies to reduce the burden of vision loss and improve patients’ quality of life. Oxidative stress and abnormal neovascularization are processes known to promote the pathological changes observed in the retina of AMD patients. The underlying molecular mechanisms triggering these processes involve aberrant downregulation of *GSTM1* and upregulation of *VEGFA* ^8, 9^. More recently, deficient levels of dietary PUFAs have shown to increase the risk of AMD and disease progression in affected individuals ^30^. Previously, our laboratory has shown that the cruciferous vegetable-derived compound, LSF, can protect human adult pigment epithelial cells from oxidative damage by upregulating *GSTM1* expression and modulating levels of selected PUFAs ^24^. Here, we validated the regenerative effects of LSF on human retinal cells under oxidative stress conditions and revealed the key fatty acids and lipids that may facilitate this protection.

A dose-dependent increase in cell proliferation was observed in LSF-treated ARPE-19 cells exposed to H _2_O _2_-induced oxidative damage but no changes in cell proliferation were detected in the absence of stress. This finding demonstrates that LSF is not harmful at the investigated micromolar doses when oxidative stress is absent but can induce regeneration of retinal cells in an oxidative stress environment. Thus, LSF may be beneficial in the treatment of AMD without causing unwanted cellular toxicity and downstream side effects.

Fatty acids are freed from the triglyceride state by a process called lipolysis. During this process, glycerol is removed from the triglycerides by lipases to release free fatty acids ^31^. The free fatty acids are then broken down to acetyl-coA in the mitochondria in the presence of nicotinamide adenine dinucleotide and the reduced form of flavin adenine dinucleotide to generate energy in a reaction known as beta oxidation ^31^. Many free fatty acids are key components of phospholipids, which stabilise the cell membranes of various cells including those of the retina. These phospholipids are cleaved into several metabolites, such as 1-palmitoyl-2-oleoyl-glycerol, which consists of side-chains derived from palmitic acid and oleic acid ^31^. Patients with AMD have demonstrated dysregulated levels of such fatty acids, which may contribute in the impairment of the retinal pigment epithelial cells seen in this disease ^32^.

To determine the types of total fatty acids possibly implicated in LSF’s impact on ARPE-19 cells, GC-MS was performed. We showed that LSF treatment increased the levels of *trans*- and *cis-*oleic acid and ETA. Oleic acid is one of the most abundant monosaturated fatty acids (MUFAs) of the omega-9 fatty acid family, while common omega-3 PUFAs include ETA, eicosapentaenoic acid and docosahexaenoic acid, found in fish oil ^33^. These fatty acids contribute to several biological processes, including visual pathways signalling in the retina, anti-inflammatory properties and protection against metabolic diseases. The benefits of a high dietary intake of omega-3 and omega-9 fatty acids in alleviating the risk of AMD by about 30% to 40% and neovascularisation have been extensively reviewed by van Leeuwen *et al.* (2018) ^30^. The action of LSF appears to be cell-type specific. Pasko *et al*. (2018) revealed that the pro-apoptotic effect of LSF on hepatocellular carcinoma and colorectal cancer cell lines was correlated with increased levels of oleic acid found in the cancer cells ^34^. This is in contrast to our findings, where no toxicity was seen in LSF-treated ARPE-19 cell line despite increased oleic acid levels. The lack of harmful effects and the evident protective effects of LSF on human retinal cells shown here can be mirrored by findings from an association study that demonstrated a correlation between a high MUFA diet and significantly reduced risk of AMD ^35^. This protective effect of MUFAs against AMD may involve anti-atherogenic pathways, as discussed by Parekh *et al.* (2009) ^35^.

Although controversial, some studies have shown that an increased dietary intake of the selected omega-3 PUFAs lowers the risk of dementia, improves cognition and aids age-related degenerative disorders ^36, 37^. Connor *et al.* used a hypoxia-induced animal model of retinopathy to show that an omega-3 PUFA diet suppressed retinal expression of the inflammatory cytokine tumour necrosis factor (TNF)-α and macrophage-induced inflammatory responses in retinal cells ^10, 38^. This anti-inflammatory phenomenon promoted a suppression of neovascularisation of comparable magnitude to that induced by VEGF inhibitors ^38, 39^. Interestingly, AMD patients demonstrated lower levels of oleic acid and omega-3 PUFAs in their red blood cells compared to their age-matched healthy controls ^32^. Furthermore, a good distribution of omega 3-PUFAs in the retina is said to be protective against photo-sensitised oxidation and peroxidation of lipids (e.g. 7-ketocholesterol) in the eyes of aging adults ^15, 32^. Oxidised lipids can induce the migration and activation of retinal microglia into an M1 pro-inflammatory phenotype, which triggers the expression of pro-angiogenic cytokines and subsequent choroid neovascularisation seen in advanced AMD. Therefore, the findings from these reports support the potential use of LSF as a naturally-occurring enhancer of omega-3 levels in RPE cells to protect RPE cells from inflammation and abnormal neovascularisation observed in AMD patients and with possibly less risk of side effects caused by conventional VEGF inhibitors ^40^. The direct relationship between the action of LSF, omega-3 PUFAs and anti-oxidative pathways has yet to be elucidated but it is known that omega-3 PUFAs, when oxidised, can protect cells against free radical superoxide and H _2_O _2 _by activating the nuclear factor erythroid-derived-2 like-2 ( *Nrf2*) pathway ^41^. It has been reported that ageing impairs Nrf2 responses to oxidative stress ^42^. As discussed in our recent publication, LSF acts as a potent Nrf-2 activator, which further promotes its use as a therapeutic agent in chronic inflammatory conditions such as AMD ^24, 43^. Future studies arising from our GC-MS data may include investigations into the possible synergistic effects of LSF and omega-3 PUFA combination treatment on the suppression of oxidative stress, neovascularisation and VEGF expression in RPE cells and choroid-derived endothelial cells.

To identify the lipid classes that are affected by LSF treatment of ARPE-19 cells, LC-MS was performed. In the presence of oxidative stress, LSF treatment decreased levels of PE lipids but increased levels of levels CE, oPDA and PC lipids. Lipofuscin, a type of pigment granule, accumulates in the ageing retina as a result of light-associated vitamin A recycling ^44^. A major component of lipofuscin is A2E, which has the capacity to destabilise cell membranes of RPE cells and compromise their viability. The creation of A2E within retinal cells involves condensation reactions between PE lipids and all-trans-retinal ^45^. The photo-oxidation of such lipids in RPE cells can be initiated via sensitisation of A2E, triggered by blue light exposure over time. Consequently, H _2_O _2_ is generated and complement is activated via C3-dependent pathways, leading to oxidative stress, inflammation and apoptosis ^46^. This supports the use of H _2_O _2 _as an ideal stimulant of both photo-oxidation and oxidative stress seen in the ageing retina of AMD patients and validates our *in vitro* model reported here. Other studies have shown that phytochemicals including anthocyanin and LSF can reduce A2E photo-oxidation and confer RPE cell protection by increasing expression of oxidative pathway phase II enzyme NAD(P)H:quinone reductase ^47^. This aligns with our previous findings where we showed that LSF treatment of ARPE-19 cells can confer protection against H _2_O _2_-induced oxidative stress by upregulating another phase II enzyme, GSTM1 ^24^. In this present study, we demonstrate that LSF treatment of ARPE-19 cells in the presence of H _2_O _2 _can downregulate levels of PE lipids (i.e. PE 34:0 and PE 38:5). Since PE lipids are precursors of A2E, we propose that retinal cells experiencing oxidative stress can benefit from LSF treatment, since this compound can reduce PE levels and, consequently, a smaller amount of PE lipids is available for the biosynthesis of A2E, which may attenuate the risk of photo-oxidation leading to retinal cell death. The levels of A2E can be measured in a further study to ascertain if the reduction of PE 34:0 and PE 38:5 can indeed reduce the levels of A2E in retinal cells.

In patients with early AMD, pathological observations include the accumulation of drusen particles containing lipoproteins in the Bruch’s membrane, accompanied by apoptosis of RPE cells. The RPE is responsible for controlling lipoprotein uptake into the retina and their distribution to photoreceptors for the replacement of shed membrane disks. These lipoproteins mainly consist of CEs but when these lipids are oxidised, they become cytotoxic to retinal cells ^48^. The levels of CEs can also be upregulated by oxidative stress stimuli, and treatment of ARPE-19 cells with lipoproteins containing oxidised lipids can increase levels of CEs consisting of oleic acid ^48^. Here, we report that LSF upregulates levels of CEs containing omega 6-PUFAs linoleic acid (18:2) and eicosadienoic acid (20:2) in the presence of H _2_O _2_. Since H _2_O _2 _is an oxidative stress stimulus, it is possible that the increased CE levels we observe in LSF-treated cells may be attributed, to some extent, to the exposure of cells to H _2_O _2_. It is noteworthy that omega 6-PUFAs are more prone to lipid peroxidation due to the increased risk of attacks to their double bonds by reactive oxygen species and because accumulation of peroxidised lipids in retinal cell membranes over time can trigger AMD progression ^35^. However, the relationship between LSF-induced mechanisms and oxidised/peroxidised lipids is not well known. Hence, a future study stemming from this work may include evaluating the oxidation/peroxidation status of lipids in LSF-treated ARPE-19 cells using well-established assays.

The vast majority of phospholipids that make up the membranes of cells in the retina are PC lipids, with omega-3 PUFAs making up about 20% of the fatty acids in this lipid class ^46^. Perhaps, the upregulation of ETA fatty acids resulting from LSF treatment observed here is reflected in the elevated levels of PC 33:3 lipids. PC and CE lipids are commonly found in drusen particles but they also accumulate in the Bruch’s membrane in normal healthy eyes throughout adulthood ^49^. Lipid accumulation in the Bruch’s membrane eventually forms a “lipid wall” that prevents the normal exchange of oxygen and nutrients between the RPE and the choroid ^15^. In addition, the higher the content of PC and CE lipids in the Bruch’s membrane, the higher the risk of lipid peroxidation and oxidation, complement activation, inflammation and generation of toxic metabolites with age. If these lipids are retained at higher levels in the RPE cells, there is a lower tendency for lipids to be shed into the Bruch’s membrane or accumulate in drusen particles; thus, lowering the risk of toxic metabolite production and apoptosis ^49^. Since LSF treatment can increase the levels of PCs and CEs in ARPE-19 cells in the current study, this suggests that this compound may have the potential to restore or maintain healthy levels of such lipids within the retinal cells by interfering with the biosynthesis or transportation of major drusen components. Genome-wide association studies have identified risk variants in genes (e.g. ATP-binding cassette transporter, cholesteryl ester transfer protein, apolipoprotein E4, etc.) that regulate lipid metabolism and transportation that may confer a protective status against AMD pathophysiology ^50^. Thus, investigating the changes in the expression of such genes may help to further dissect the lipid pathways responsible for the LSF-mediated regeneration of RPE cells under oxidative stress conditions.

 Lipids are major components of plant stress hormones. An example is oPDA, which is the key precursor of the oxylipin stress hormone, jasmonate. oPDA lipids can activate genes involved in oxidative stress pathways and a correlation between oPDA signalling and decreased hydrogen peroxide levels has been reported in plants ^51, 52^. Interestingly, administrating the stress hormone jasmonate to broccoli sprouts increased levels of LSF ^53^. This suggests that LSF may be a by-product of a compensatory mechanism found in plants that maintains cellular redox homeostasis in stressful environments. Additionally, oPDA treatment of human neuroblastoma cells can prevent harmful effects from oxidative stress and apoptosis by activating the Nrf2 pathway ^54^. The redox activity of oPDA is also evidenced in its capacity to regulate the expression of *GST* genes ^55^. Taken together, oPDA behaves as a Nrf2 activator like LSF. Therefore, LSF’s antioxidant effects on ARPE-19 cells shown here may either involve: 1) independent activation of Nrf2; 2) an upregulation of oPDA, which in turn triggers the Nrf2 pathway; or 3) a synergistic activation of this pathway mediated by the combined action of LSF and oPDA signalling.

Although we attempt to discuss the possible relationship between the observations arisen from the total fatty acid analysis (GC-MS) and lipidomic data (LC-MS), drawing a correlation between fatty acid data and the LC-MS lipid profile in this study proved to be challenging, since the methods used here could not explicitly identify the source of the fatty acids (i.e. free/circulating or conjugated to lipids) implicated in LSF’s protection of the ARPE-19 cell line. In addition, the metabolomics data here was first normalised to the internal standard d7-cholesterol to monitor the variations of the extraction efficiency and then further normalised to cell numbers since the cell numbers varied between replicates. It is important to note that the raw data exported using Mass Hunter Quant V6 software will not show the d7-cholesterol peak area and hence is not presented in this manuscript. Despite these limitations, this study revealed the ability of LSF to alter levels of selected fatty acids and lipid classes involved in mechanisms that can promote AMD processes in human RPE cells.

In conclusion, we propose that RPE cells at risk of apoptosis can be pre-conditioned with LSF to regulate levels of selected fatty acids and lipids known to be implicated in downstream pathways of photo-oxidation, inflammation and oxidative stress for the generation of a protective state against the ageing process and AMD progression. This work warrants future investigations, such as trialling LSF treatment in co-culture models of ARPE-19 and choroid-derived cells, and animal models of AMD. Performing high throughput transcriptomics methods will also help to identify key genes that mediate LSF’s effects on fatty acid and lipid metabolism, biosynthesis and translocation in RPE cells under AMD-like pathological conditions ^56^. Other future experiments such Annexin V apoptosis detection and TNF-α assays that can demonstrate LSF’s protection against apoptosis and lipidomic response will also be beneficial. These further studies will facilitate the design of targeted therapies that can be co-administered with LSF for the management of AMD progression.

## Data availability

### Underlying data

Harvard Dataverse: Lipidomics reveal the protective effects of a vegetable-derived isothiocyanate against retinal degeneration. https://doi.org/10.7910/DVN/C9VCBX ^28^

This project contains the following underlying data:

- GCMS Fatty Acid Analysis Data.tab (raw fatty acid analysis data)
- LCMS Lipid Analysis Data.tab (raw lipid analysis data)
- MTS Raw Data_Kwa.tab (raw cell proliferation assay data)
- Cell Count Data for Metabolomics

Data are available under the terms of the Creative Commons Zero “No rights reserved” data waiver (CC0 1.0 Public domain dedication).
