# Supplementary material for: Lipidomics reveal the protective effects of a vegetable-derived isothiocyanate against retinal degeneration
Source: F1000Res. 2020 Oct 5;8:1067. Originally published 2019 Jul 12. [Version 5] doi: 10.12688/f1000research.19598.5 (PMC7590896; doi:10.12688/f1000research.19598.5)
Supplement: Peer review history

# Peer review: Reviewer response for version 5

**DOI:** 10.5256/f1000research.29839.r72434

**Published:** 2020-10-26

**Authors:** Timothy Couttas

**Affiliations:** 1 University of Sydney, Sydney, Australia

No additional comments.

Is the work clearly and accurately presented and does it cite the current literature?

Yes

If applicable, is the statistical analysis and its interpretation appropriate?

Partly

Are all the source data underlying the results available to ensure full reproducibility?

Partly

Is the study design appropriate and is the work technically sound?

Partly

Are the conclusions drawn adequately supported by the results?

No

Are sufficient details of methods and analysis provided to allow replication by others?

Partly

Reviewer Expertise:

NA

I confirm that I have read this submission and believe that I have an appropriate level of expertise to confirm that it is of an acceptable scientific standard.

# Peer review: Reviewer response for version 5

**DOI:** 10.5256/f1000research.29839.r72433

**Published:** 2020-10-22

**Authors:** Alexa Klettner

**Affiliations:** 1 Department of Ophthalmology, University of Kiel, Quincke Research Center, Haus 25, Kiel, 24105, Germany

My concerns have been addressed by the authors and I now approve the publication.

Is the work clearly and accurately presented and does it cite the current literature?

Yes

If applicable, is the statistical analysis and its interpretation appropriate?

Yes

Are all the source data underlying the results available to ensure full reproducibility?

Yes

Is the study design appropriate and is the work technically sound?

Partly

Are the conclusions drawn adequately supported by the results?

No

Are sufficient details of methods and analysis provided to allow replication by others?

Yes

Reviewer Expertise:

experimental retinology; RPE cell culture; age-related macular degeneration (pathogenesis and therapy)

I confirm that I have read this submission and believe that I have an appropriate level of expertise to confirm that it is of an acceptable scientific standard.

# Peer review: Reviewer response for version 5

**DOI:** 10.5256/f1000research.29839.r72435

**Published:** 2020-10-08

**Authors:** Yanfei (Jacob) Qi

**Affiliations:** 1 Centenary Institute, University of Sydney, Camperdown, NSW, Australia

No further comments.

Is the work clearly and accurately presented and does it cite the current literature?

Partly

If applicable, is the statistical analysis and its interpretation appropriate?

Partly

Are all the source data underlying the results available to ensure full reproducibility?

Yes

Is the study design appropriate and is the work technically sound?

Partly

Are the conclusions drawn adequately supported by the results?

Partly

Are sufficient details of methods and analysis provided to allow replication by others?

Yes

Reviewer Expertise:

Cell biology, lipid metabolism and lipidomics

I confirm that I have read this submission and believe that I have an appropriate level of expertise to confirm that it is of an acceptable scientific standard.

# Peer review: Reviewer response for version 4

**DOI:** 10.5256/f1000research.28220.r68618

**Published:** 2020-08-27

**Authors:** Alexa Klettner

**Affiliations:** 1 Department of Ophthalmology, University of Kiel, Quincke Research Center, Haus 25, Kiel, 24105, Germany

The report as been revised mostly according to the reviewers suggestions. A few points, however, remain:

 Introduction:

 Wet AMD is one of the late stages of AMD, not the only late stage. This needs to be corrected.

 Results:

 The degree of cell death induced by the treatment of H2O2 has to be given in the result section. Even though the effect of H2O2 has been published before, this needs to be shown for the current set of experiments.

Is the work clearly and accurately presented and does it cite the current literature?

Yes

If applicable, is the statistical analysis and its interpretation appropriate?

Yes

Are all the source data underlying the results available to ensure full reproducibility?

Yes

Is the study design appropriate and is the work technically sound?

Partly

Are the conclusions drawn adequately supported by the results?

No

Are sufficient details of methods and analysis provided to allow replication by others?

Yes

Reviewer Expertise:

experimental retinology; RPE cell culture; age-related macular degeneration (pathogenesis and therapy)

I confirm that I have read this submission and believe that I have an appropriate level of expertise to confirm that it is of an acceptable scientific standard, however I have significant reservations, as outlined above.

# Response

**Published:** 2020-09-23

**Authors:** Faith Kwa

**Affiliations:** Swinburne University of Technology, Australia

We thank Professor Klettner for her further comments dated 27 August 2020. To address her final two comments, we have made the following changes which are now incorporated into Version 5 of the manuscript. 

**Response to "Introduction: Wet AMD is one of the late stages of AMD, not the only late stage. This needs to be corrected": **We have now amended the statement in the Introduction and it now reads: "In one of the late stages of AMD (wet AMD), the atrophic retinal tissue becomes replaced with granulation tissue consisting of abnormal leaky blood vessels". 

**Response to "Results: The degree of cell death induced by the treatment of H2O2 has to be given in the result section. Even though the effect of H2O2 has been published before, this needs to be shown for the current set of experiments":** Figure 1 which shows the net effect of hydrogen peroxide on the proliferation of ARPE-19 cells have been added to Version 5 of the manuscript. This figure is now accompanied by a description of these observations in the Results section. These observations show a consistent trend previously published in our earlier article.

We believe that the changes reflected in Version 5 of the manuscript address the remaining points Professor Klettner has raised. We kindly request Professor Klettner to approve our manuscript unconditionally.

We would also like to point out to our reviewers that a final decision can be made to approve this manuscript without having to add any further comments. Also, any questions which Reviewers post in response to my further comments which may not necessarily warrant any changes to the current version will mean that a sixth version need to be published in order for reviewers to make a decision again.

# Peer review: Reviewer response for version 4

**DOI:** 10.5256/f1000research.28220.r68616

**Published:** 2020-08-17

**Authors:** Timothy Couttas

**Affiliations:** 1 University of Sydney, Sydney, Australia

1. Cut-off for signal to noise ratio - The underlying data reports several lipids with a very low peak area response (For example PI 32:0 reports a value in the untreated group as 78). I am concerned that some of their lipid identifications have been falsely identified. Does metaboanalyst have a threshold for considering ‘real’ peaks compared to noise?
2. Accounting for additional internal standards in the underlying data – The authors have clarified in the discussion the use of cholesterol d7 to normalise their results. However, the underlying data presents several other internal standards as being present (for example LPC 19:0, PI 38:4, SM 30:1, TG 17:0/17:0/17:0) We’re these internals standards also present, and if so why weren’t they used to normalise their respective lipid classes, and if not have they simply been falsely identified?
3. Statistical analysis - As there was no direct response to my initial query I am still unsure of the rationale behind Bonferroni over FDR/Benjamini-Hochberg. To the best of my knowledge, both are options for correcting P-values using metaboanalyst with FDR . Although not affecting the fold changes observed, FDR has more power and would help clarify the more statistically significant changes.

Is the work clearly and accurately presented and does it cite the current literature?

Yes

If applicable, is the statistical analysis and its interpretation appropriate?

Partly

Are all the source data underlying the results available to ensure full reproducibility?

Partly

Is the study design appropriate and is the work technically sound?

Partly

Are the conclusions drawn adequately supported by the results?

No

Are sufficient details of methods and analysis provided to allow replication by others?

Partly

Reviewer Expertise:

NA

I confirm that I have read this submission and believe that I have an appropriate level of expertise to confirm that it is of an acceptable scientific standard, however I have significant reservations, as outlined above.

# Response

**Published:** 2020-08-26

**Authors:** Faith Kwa

**Affiliations:** Swinburne University of Technology, Australia

We thank Dr Timothy Couttas for his comments. Please see our response to each of his three points. We believe that these have addressed his comments and we hope Dr. Couttas will unconditionally approve our article. Thank you.

**Response to Point 1: **All the peaks were manually checked for the integration including the low peak area. Also,  MetaboAnalyst was used to perform statistical analysis, to find out significant differences in between the related groups. If the real peaks are closed to noise or not detected in one group, MetaboAnalyst software will provide the significant differences with the consideration of the standard deviation of the replicates within the group. That will eliminate the falsely identified peaks reporting as significant peaks.

**Response to Point 2:** We used d7-cholesterol as an internal standard to monitor the variations of the extraction efficiency only. As we have planned to compare the lipid profile changes, rather than absolute lipid concentration, we did not use internal standards for each lipid class for semi-quantification of the lipids.

**Response to Point 3:**  In our study, the Bonferroni method which is a standard p-value correction tool provided in MetaboAnalyst was used. This method is routinely used by researchers in the metabolomics community and it is a single step procedure, where each p-value is corrected independently. The p-value of each compound is multiplied by the number of compounds in the compound list. If the corrected p-value is still below the error rate, the compound will be significant.  Corrected P-value= p-value * n (number of compounds in test) <0.05. As a consequence, if we say test 1000 compounds at a time, the highest accepted individual p-value is 0.00005, making the correction very stringent. With a Family-wise error rate of 0.05 (i.e., the probability of at least one error in the family), the expected number of false positives will be 0.05.  Therefore, Bonferroni method provides the strongest control of the false positives and therefore a high confidence in the selected metabolic features particularly for the purposes of novel untargeted studies where new metabolites are identified in the context of the research they are conducted in. Some researchers in the field of metabolomics also advocate that the Bonferroni method  is both conceptually easier to understand and numerically easier to implement than FDR methods such as those described by Yoav Benjamini and Yosef Hochberg (see https://link.springer.com/article/10.1007/s11306-006-0037-z).  FDR methods compute the number of false positives out of the significantly varied metabolic features, (i.e., the rate of significant features being false). This is different from the Bonferroni correction which focuses on the control on all falsely rejected hypotheses. For the above reasons, we have selected Bonferroni over FDR/Benjamini-Hochberg.

# Peer review: Reviewer response for version 3

**DOI:** 10.5256/f1000research.24763.r65473

**Published:** 2020-07-09

**Authors:** Timothy Couttas

**Affiliations:** 1 University of Sydney, Sydney, Australia

In this manuscript, Kwa *et al* aim to build on their prior work, demonstrating that pre-treatment of ARPE-19 cells with L-Sulforaphane (LSF) protects against hydrogen peroxide induced oxidative damage. In their current study, Kwa *et al* have employed GC and LC-MS to the analysis of fatty acids and lipids, in order to ascertain the lipid pathways that infer LSF protection in their in vitro model of age-related macular degeneration (AMD).

 Kwa *et al* report that in response to LSF treatment several alterations occur to fatty acids oleic acid and eicosatrienoic acid, as well as several lipids classes (PC, PE, CE) perturbed under oxidative stress following pre-treatment with LSF. 

 Although this manuscript demonstrates how altered lipid metabolism may be implicated in the mechanism of protection for LSF in AMD, there are inconsistencies and limitations in their LC-MS/MS method, processing of the lipidomic data, statistical analysis, and interpretation of the findings which need to be addressed and/or clarified before this manuscript is suitable for indexing.

**Major queries**

Processing of lipidomic data

 Kwa *et al* state that “The lipidomic data was generated using reverse phase **peak area response**of each lipid series rather than absolute concentrations. Did the authors employ internal standards prior to their lipid extraction?

 In complex samples, it is fairly common for variability to occur in the ionisation of lipid ions, even from repeat measures of the same sample. The use of an internal standard allows us to control for that variability, as well as any variations in the extraction (particularly when a 2-phase extraction like CHCL3/MeOH is employed) and HPLC injection. In addition, when employing reverse phase LC-MS, multiple internal standards per lipid class may be required, as lipids are often spread across a wide retention time. The class of lipid can also influence the ionisation efficiency, as it has been previously demonstrated that different lipid standards spiked into samples, at the same concentration, can have up to orders of magnitude differences in intensities (PMID: 11290839) ^1^. This can lead to differing ion suppression effects over the course of the LC run, particularly if a gradient LC was employed (PMID: 31820027) ^2^.

 In the underlying data there appears to be several internal standards listed (IS): LPC 19:0 IS, PI 38:4 IS, SM 30:1 IS, LPC 19:0 IS, Cer 17:0). Why have the authors only provided the peak areas in the underlying data and not the values normalised to these internal standards?

 If these internal standards were used, they should be included in the text and the underlying data adjusted. If not, then this may have severe implications of the data obtained and needs to be discussed as a limitation of this study.

 In the underlying data, there were also several incidences of lipid IDs with very low peak area response (e.g. CE 14:0, LPC 22:0). How was the limit of detection defined against the background noise acquired (i.e. signal to noise ratio). There may be incidences of false positive IDs for lipids at trace levels, which may influence the statistical significance of the results.

Statistical analysis and presentation of underlying data

 Kwa *et al* report that significant changes in lipid levels of 20μM LSF treatment groups in the presence of oxidative stress, which were derived from paired t-test, along with one-way ANOVA with post-hoc Bonferroni analysis, using MetaboAnalyst.    

 With a large number of lipid identities, there is an increasing risk of number of false-positive identities owing to multiple testing. The authors have corrected for this using the Bonferroni multiple testing correction technique in MetaboAnalyst. However the Benjamini–Hochberg correction or false discovery rate (FDR) is often recommended as it can control for the proportion of false positives amongst all significant results.

 As both FDR- and Bonferroni-corrected P-values are provided by MetaboAnalyst, could the authors please provide an explanation as to why FDR wasn’t used or update the results to show the impact of FDR on their significant lipid changes? I would also recommend reporting the p-values for all lipid comparisons in their underlying data and not just the raw values.

Interpretation of lipidomic data

 Kwa *et al* propose that their cellular model implicates RPE cells, at risk of oxidative damage and apoptosis, can be pre-conditioned with LSF for the generation of a protective state against age-related AMD. However, their results state that LSF treatment only induced significant alterations to lipids (decreased PE and increases in CE, oPDA and PC lipids) when in the presence of oxidative stress. Could the lipidomic changes observed simply be as a consequence of hydrogen peroxide-induced oxidative stress?

 The authors do make the acknowledgement that “ *Since H2O2 is an oxidative stress stimulus, it is possible that the increased CE levels we observe in LSF-treated cells may be attributed, to some extent, to the exposure of cells to H2O2*”. However I think this statement should also reflect plausible reasoning for the elevated levels of PC 33:3 and oPDA 34:3-PC 16:0, which upon review of the underlying data, also display elevated levels in the untreated and 5mM LSF groups, when exposed to hydrogen peroxide. This however may not be the case in the normalised data, but as this data was not included in the underlying data I cannot evaluate whether this is true.

 In the underlying data, the 20mM LSF +H2O2 group display the most significant elevated levels of CE, PC 33:3 and oPDA 34:3-PC 16:0, compared to the rest of the groups. However without normalised data (to appropriate internal standards) you cannot resolve whether this is simply due the variation in intensities of the peak area data presented.

 Could the authors also please clarify whether the untreated controls in Figure 5 are exposed to H2O2 or not? When reviewing the underlying data, there is considerable variability in the untreated (-H2O2) results for PE 34:0 (with peak areas in the underlying file ranging from 25,000 to 5000).

 I would also suggest that the authors either consider the limitation of their proposed model for LSF conditioning in their discussion or provide additional experiments/future work demonstrating LSF protection against apoptosis and lipidomic response (e.g. protection against TNFα-induced apoptosis; PMID: 29548329 ^3^).

**Additional comments**

 Kwa *et al* report that *“Lipid analysis using LC-MS was carried out as published previously (Ref - 26)”*. However the reference in question “Olmstead IL et al: *A quantitative analysis of microalgal lipids for optimization of biodiesel and omega-3 production*.” does not provide the necessary methodology with which to repeat the LC-MS analysis. I believe the authors may have simply cited the wrong reference. The prior reference in their manuscript (Botte CY et al.: “ *Atypical lipid composition in the purified relict plastid (apicoplast) of malaria parasites*”) contains the relevant supporting information for LC-MS analysis, including reverse-phase chromatographic separation and MRM precursor-product ion pairs for mass spectrometry. 

 Proper assignment of sphingolipids should be reported in their underlying data using the LipidMAPS ( http://www.lipidmaps.org) nomenclature system. This will be based on the MRM transitions used. If the authors were screening for the 264 m/z product ion of sphingolipids, this insinuates that the sphingolipid identified contains a sphingosine backbone (d18:1). Hence ceramides should be written accordingly (e.g. Cer 24:0 should be written as Cer (d18:1/24:0).

 Your cell model compared the proliferative effects of treated and untreated cells in the presence and absence of H2O2 - separately. Were there any differences in the proliferation rate when untreated cells in the absence of H2O2 were compared to LSF treatment with H2O2? Does presence of LSF restore or maintain normal cell proliferation when cells are exposed to H2O2?

 In their discussion, Kwa et al state that “ *Many studies have shown that an increased dietary intake of the selected omega-3 PUFAs lowers the risk of dementia, improves cognition and aids age-related degenerative disorders*”. There is conflicting evidence whether this is indeed true, which the authors should state in their discussion. In fact the study cited by the authors actually states that their *“results do not provide evidence of a beneficial effect of fish-oil supplementation on cognitive function….”.*

Is the work clearly and accurately presented and does it cite the current literature?

Yes

If applicable, is the statistical analysis and its interpretation appropriate?

Partly

Are all the source data underlying the results available to ensure full reproducibility?

Partly

Is the study design appropriate and is the work technically sound?

Partly

Are the conclusions drawn adequately supported by the results?

No

Are sufficient details of methods and analysis provided to allow replication by others?

Partly

Reviewer Expertise:

Lipid metabolism, Lipidomics, LC-MS/MS

I confirm that I have read this submission and believe that I have an appropriate level of expertise to state that I do not consider it to be of an acceptable scientific standard, for reasons outlined above.

## References

[1] : Quantitative determination of phospholipid compositions by ESI-MS: effects of acyl chain length, unsaturation, and lipid concentration on instrument response. *J Lipid Res*.2001;42(4) :663-72 11290839

[2] : Lipidomics from sample preparation to data analysis: a primer. *Anal Bioanal Chem*.2020;412(10) : 10.1007/s00216-019-02241-y 2191-2209 10.1007/s00216-019-02241-y 31820027PMC7118050

[3] : Chronic exposure to tumor necrosis factor alpha induces retinal pigment epithelium cell dedifferentiation. *J Neuroinflammation*.2018;15(1) : 10.1186/s12974-018-1106-8 85 10.1186/s12974-018-1106-8 29548329PMC5857126

# Peer review: Reviewer response for version 3

**DOI:** 10.5256/f1000research.24763.r65469

**Published:** 2020-07-06

**Authors:** Alexa Klettner

**Affiliations:** 1 Department of Ophthalmology, University of Kiel, Quincke Research Center, Haus 25, Kiel, 24105, Germany

In their study, the authors investigate the effect of L-sulforaphane (LSF) on the lipid distribution and proliferation of ARPE-19 cells with the conclusion that LSF changes the lipid distribution and induces proliferation. While the study is of interest, especially as lipids are a major yet understudied aspect of age-related macular degeneration, there are some major concerns. The most pressing concern is that the claims made by the authors concerning the effect on proliferation are not really shown by the data.

**Introduction**

 The claims make in the introduction are not always accurate. The authors state that “each of these treatments resulted in the development of atrophic scars and haemorrhage”. This is not correct. Haemorrhage is very seldom found after anti-VEGF treatment. Also, while atrophic degenerations (not "scars") are found in patients treated with anti-VEGF treatment, it is still under debate whether there are because of in spite of the treatment.

 The authors also state that AMD “is known to be regulated by genes such as VEGFA or GSTM1”. This is imprecise. When it comes to risk factors, the most important genes are of the complement system.The secreted VEGF-A protein is very important for late exudative AMD development. A more careful wording is requested.

**Methods**

 Please include briefly in the appropriate subsection what exactly is measured in the FAME analysis.

**Results**

 The authors claim that LSF incudes proliferation in ARPE-19 cells after H _2_O _2 _ treatment, shown in MTS assay. This conclusion is problematic, as the authors to not actually show the proliferation. The same effect seen in the results could be the result of a protective effect of LSF against oxidative stress induced cell death. Unfortunately, the authors do not show to what extend cell death is induced by H _2_O _2_, as they use the H _2_O _2 _treated cells as the 100% control. This is not appropriate, the MTS should be normalized to untreated cells. (The authors claim that they have already shown cell death by H _2_O _2_ in another paper. This is actually irrelevant for this point, as the control is not chosen correcty and the claim of proliferation is not substantiated by the data.)

 As this effect of LSF is an important claim of the paper, I also suggest perform additional experiments to investigate whether it is a protection from cell death or an increase proliferation that is induced by LSF.

**Figures**

 It is not clear in the figures 4 and 5 if “untreated” means untreated cells or cells treated with H _2_O _2_ only. This needs to be clarified.

 In Figure 2 and 4, please give an explanation for the abbreviations used in the legend.

**Discussion**

 I would strongly suggest concentrating the discussion on the findings of the study. E.g., drusen are not really a topic of this study.

 Also, the authors should discuss the limitation of their model. ARPE-19 cells model aspects of RPE cells. Please elaborate whether ARPE-19 are similar to RPE cells in regard to lipids.

Is the work clearly and accurately presented and does it cite the current literature?

Yes

If applicable, is the statistical analysis and its interpretation appropriate?

Yes

Are all the source data underlying the results available to ensure full reproducibility?

Yes

Is the study design appropriate and is the work technically sound?

Partly

Are the conclusions drawn adequately supported by the results?

No

Are sufficient details of methods and analysis provided to allow replication by others?

Yes

Reviewer Expertise:

experimental retinology; RPE cell culture; age-related macular degeneration (pathogenesis and therapy)

I confirm that I have read this submission and believe that I have an appropriate level of expertise to confirm that it is of an acceptable scientific standard, however I have significant reservations, as outlined above.

# Response

**Published:** 2020-07-20

**Authors:** Faith Kwa

**Affiliations:** Swinburne University of Technology, Australia

In their study, the authors investigate the effect of L-sulforaphane (LSF) on the lipid distribution and proliferation of ARPE-19 cells with the conclusion that LSF changes the lipid distribution and induces proliferation. While the study is of interest, especially as lipids are a major yet understudied aspect of age-related macular degeneration, there are some major concerns. The most pressing concern is that the claims made by the authors concerning the effect on proliferation are not really shown by the data.

**RESPONSE**: We thank Professor Klettner for her constructive comments and have made the amendments and rebuttal accordingly. 

**Introduction**

 The claims make in the introduction are not always accurate. The authors state that “each of these treatments resulted in the development of atrophic scars and haemorrhage”. This is not correct. Haemorrhage is very seldom found after anti-VEGF treatment. Also, while atrophic degenerations (not "scars") are found in patients treated with anti-VEGF treatment, it is still under debate whether there are because of in spite of the treatment.

**RESPONSE**: We thank Professor Klettner for pointing this out. We have now reworded the paragraph to read: “Conventional therapies include FDA-approved anti-angiogenic agents, thermal laser photocoagulation or intravitreal injection of medications to limit neovascularisation (8). However, each of these treatments do not cure AMD but mainly reduce patients’ symptoms and usually target the late stages of the disease where abnormal neovacularisation is observed”.

 The authors also state that AMD “is known to be regulated by genes such as VEGFA or GSTM1”. This is imprecise. When it comes to risk factors, the most important genes are of the complement system.The secreted VEGF-A protein is very important for late exudative AMD development. A more careful wording is requested.

**RESPONSE**: We thank Professor Klettner for pointing this out too. We have now reworded the paragraph to read “In oxidative stress conditions including those observed in the retina of patients with early stage ‘dry’ AMD, redox enzymes coded by genes such as *glutathione-S-transferase µ1* ( *GSTM1*) are important in preventing ROS accumulation in the retina but these genes are often expressed in low levels in AMD patients (2). In later stages of AMD (i.e. ‘exudative’ AMD), genes such as *Vascular Endothelium Growth Factor A* ( *VEGFA*) play a vital role in its development (8, 9)”.

**Methods**

 Please include briefly in the appropriate subsection what exactly is measured in the FAME analysis.

**RESPONSE**: As mentioned in the Methods section, “Extraction of fatty acids and lipids from treated ARPE-19 Cells for GC-MS and LC-MS analysis”, ‘FAME’ stands for fatty acid methyl ester. However to improve the clarify regarding FAME analysis, we have now added the following sentence under “FAME analysis using GC-MS” in the Methods section,  “The FAME analysis which measure the level of fatty acids in each sample was carried out using a 30 m column with a 0.25 μm film thickness, 0.25 mm inner diameter and a 10 m guard column (catalogue number CP8944, Agilent J&W Scientific VF-5MS GC Column)”.

**Results**

 The authors claim that LSF incudes proliferation in ARPE-19 cells after H _2_O _2 _ treatment, shown in MTS assay. This conclusion is problematic, as the authors to not actually show the proliferation. The same effect seen in the results could be the result of a protective effect of LSF against oxidative stress induced cell death. Unfortunately, the authors do not show to what extend cell death is induced by H _2_O _2_, as they use the H _2_O _2 _treated cells as the 100% control. This is not appropriate, the MTS should be normalized to untreated cells. (The authors claim that they have already shown cell death by H _2_O _2_ in another paper. This is actually irrelevant for this point, as the control is not chosen correcty and the claim of proliferation is not substantiated by the data.)

 As this effect of LSF is an important claim of the paper, I also suggest perform additional experiments to investigate whether it is a protection from cell death or an increase proliferation that is induced by LSF.

**RESPONSE**: The MTS assay used in this research is a well-established cell proliferation assay that is used widely by many, even in recent times (e.g. https://pubmed.ncbi.nlm.nih.gov/30868895/, https://pubmed.ncbi.nlm.nih.gov/30242861/and https://pubmed.ncbi.nlm.nih.gov/32219038/). We used the formula recommended by the manufacturer Promega’s protocol to calculate the % cell proliferation which involves normalising the absorbance values  LSF-treated cells against the control (i.e untreated cells) in the respective normal conditions (- H _2_O _2 _) and oxidative stress conditions (+H _2_O _2 _). The term “untreated cells” refer to cells not treated with LSF regardless of the oxidative stress stimulus being present or not. This is now added into the Methods section, “Cell Treatment Prior to Analysis”. We have also conducted 4 replicates per treatment condition and independently repeated the experiment 3 times to generate Figure 1. The aim of Figure 1 is to illustrate the net effect of LSF on the retinal cells with and without oxidative stress. This is to show that under normal conditions, LSF is not toxic to retinal cells unlike what is observed in cancer cells *in vitro* as published by others referenced in the Introduction section of the manuscript. In contrast, LSF increased cell proliferation when the cells were exposed to H _2_O _2 _ when compared to cells without LSF treatment (i.e. untreated) under the same oxidative stress conditions. Normalising LSF-treated cells +  H _2_O _2 _ against untreated cells -  H _2_O _2 _ as suggested is not ideal as there will be 2 confounding variables (absence/presence of LSF and H _2_O _2 _) here which will not allow us determine the net effect of LSF on the cells under normal and oxidative stress conditions respectively. In view of the above, the conclusion that LSF can promote cell proliferation under oxidative stress conditions reported in this manuscript is valid. To avoid any indication that LSF inhibits apoptosis in retinal cells, we have also now where applicable in the appropriate context, replace the term “protective” with “regenerative” as tissue regeneration is brought about by cell proliferation. However, in the context of oxidative stress, we have indeed showed in our previous study (Reference 24) that LSF has the potential to protect in the retinal cells against oxidative stress by increasing levels of the gene that encodes redox enzyme GSTM1;  and the rationale of the study of fatty acids and lipids here is linked to their role in oxidative stress-related pathways as stated in the Introduction of the manuscript.

 The cells used in the experiments reported in this paper were from the same batch of cells used in our previous paper (24). Given that the net effect of hydrogen peroxide has already been reported previously, it is not novel to present this data again in this publication and readers may refer to the reference provided just like how many authors refer to their previous work when detailing methodologies previously used.

**Figures**

 It is not clear in the figures 4 and 5 if “untreated” means untreated cells or cells treated with H _2_O _2_ only. This needs to be clarified. In Figure 2 and 4, please give an explanation for the abbreviations used in the legend.

**RESPONSE**: The term “untreated cells” refer to cells not treated with LSF regardless of the oxidative stress stimulus being present or not. This definition is now added under “Cell treatment prior to analysis” in the Methods Section.  All definitions of abbreviated terms for the fatty acids and lipids that are consistent in their changes in the levels across the replicates or are shown to be statistically significantly different in their levels between treated groups and untreated controls (i.e fatty acids and lipids highlighted in the red boxes in Figures 2 and 4 respectively) have been listed in the Figure legends. However, for other non-significant fatty acids and lipids shown in the heatmap which are not discussed in this manuscript, these are not explained in the legends as this would cause a non-exhaustive list of definitions. For readers who are less familiar with nomenclature for lipids and fatty acids, they can refer to this statement already in the manuscript under “Lipid Analysis using LC-MS”: “Detected lipid species were annotated as lipid class (sum of carbon atoms in the  fatty acid chains:sum of double bonds in the fatty acid chains)”. In addition,  we have now added the statement “Detected fatty acids were annotated as: first two digits as sum of carbon atoms in the  fatty acid chains followed by a single digit that indicates the sum of double bonds in the fatty acid chains.” under “FAME Analysis using GC-MS”. For more information, kindly refer to https://www.lipidmaps.org/resources/tutorials/lipid_cns.html

**Discussion**

 I would strongly suggest concentrating the discussion on the findings of the study. E.g., drusen are not really a topic of this study. Also, the authors should discuss the limitation of their model. ARPE-19 cells model aspects of RPE cells. Please elaborate whether ARPE-19 are similar to RPE cells in regard to lipids.

**RESPONSE**: The results have been discussed in detail in the Discussion section and how the findings could potentially relate to the AMD context. As this area of research is relatively niche and there are not many other publications that report PC and CE lipids in AMD patients, we could only discuss the observations related to PC and CE lipid levels based on publications reporting these lipid classes as components of drusen.  We do not claim that LSF can prevent drusen formation and we do list that not being able to draw a direct relationship between our fatty acid and lipid data is a limitation that warrants further studies such as transcriptomic analysis which could well include determining the expression of the Clusterin gene which codes for proteins present in drusen.

 The human cell line, ARPE-19, used in these experiments is derived from the retinal pigmented epithelium of a 19 year old male ( https://www.atcc.org/products/all/CRL-2302.aspx#) which is from the same batch of cells used in our previous study Reference 24. Cell characterisation had been reported there. An image of the cells and confocal validation of the cell line using Human Adult Pigment Epithelial cells–19 (ARPE-19) and human epithelial colorectal adenocarcinoma cells (Caco-2) as negative control cell line stained with the Hoechst nuclear dye (blue) and the anti-cellular retinal-binding protein (CRALBP) antibody were provided.  Reference to Reference 24 has already been made in “Cell Culture” of the Methods section. Furthermore, one study ( https://pubmed.ncbi.nlm.nih.gov/29097185/) has shown that serum deprivation in ARPE-19 cultures  mimics the pattern of cholesterol distribution see in AMD while another study reported that ARPE-19 cells resemble an ageing RPE ( https://iovs.arvojournals.org/article.aspx?articleid=2186765). Therefore, the lipid profile of the ARPE-19 cells used here would closely reflect those of the RPE.

 We have addressed all the comments raised by Professor Klettner and hope that our article can now be approved without reservations.

# Peer review: Reviewer response for version 3

**DOI:** 10.5256/f1000research.24763.r60336

**Published:** 2020-03-25

**Authors:** Yanfei (Jacob) Qi

**Affiliations:** 1 Centenary Institute, University of Sydney, Camperdown, NSW, Australia

Following up my questions in the previous review:

 Q1 and Q2: I fully understand the in vitro model of H2O2-induced oxidative stress as well as the related cell biology assays. If the Fig 1A and 1B are combined together, readers can see the trend upon treatments, while also comparing the information under the -/+ H2O2 conditions.

 Q3: I mean +LSF-H2O2 vs -LSF+H2O2, they caused similar changes in the levels of C18:1 and C20:3, according to the Underlying Data. Why did these two treatments resulted in opposite biological outcomes, if C18:1 and C20:3 are key regulators?

 Q4: Based on your results, another 19 types of PEs are more abundant than 34:0 by the analyses of Prism. I quite understand that PE34:0 is not the LEAST abundant, as many others like 34:3 are even lower. In my analyses, the total PE level was significantly reduced in LSF20+H2O2, as compared with -LSF+H2O2 or LSF5+H2O2. This might be more related to your discussion on A2E production.

Is the work clearly and accurately presented and does it cite the current literature?

Partly

If applicable, is the statistical analysis and its interpretation appropriate?

Partly

Are all the source data underlying the results available to ensure full reproducibility?

Yes

Is the study design appropriate and is the work technically sound?

Partly

Are the conclusions drawn adequately supported by the results?

Partly

Are sufficient details of methods and analysis provided to allow replication by others?

Yes

Reviewer Expertise:

Cell biology, lipid metabolism and lipidomics

I confirm that I have read this submission and believe that I have an appropriate level of expertise to confirm that it is of an acceptable scientific standard, however I have significant reservations, as outlined above.

# Response

**Published:** 2020-04-15

**Authors:** Faith Kwa

**Affiliations:** RMIT University, Australia

*Q1 and Q2: I fully understand the in vitro model of H2O2-induced oxidative stress as well as the related cell biology assays. If the Fig 1A and 1B are combined together, readers can see the trend upon treatments, while also comparing the information under the -/+ H2O2 conditions.*

**Response:** We thank Reviewer 2 for his further comments. As mentioned before, the purpose of splitting the data into -/+ H2O2 are to assess the net effects of LSF on the cells under normal conditions and oxidative stress conditions respectively.  Therefore, for the consistency of data presentation throughout the manuscript, all data reported have been presented in this format. Combining the data will result in depicting the effects of hydrogen peroxide on the cell line rather than the effects of LSF in conditions with or without oxidative stress stimulus. Furthermore, the data analysis relating to MTS assay as specified by the Manufacturer requires normalisation to a control such that if we were to combine the data and plot it on the same graph, all the -H202 treatment conditions (LSF -H202, DMSO -H202 and untreated -H202) will all be depicted as 100% according to the manufacturer’s formula due to the normalisation. We would like to respectfully point out that this is not aim of the Figure 1. Comparing + H202 and -H202 groups will only highlight the effect of H202 on the cell line rather than the effect of LSF under normal and oxidative stress conditions. We have previously published the net effect of hydrogen peroxide on the ARPE-19 cell line: https://www.ncbi.nlm.nih.gov/pubmed/29376497 . In this earlier publication, we have shown that the 200µM H202 following a 2 hour exposure reduced % cell proliferation by 61.2% (p < 0.001). Therefore, we hope that the Review can understand why Figure 1 remains unchanged.

*Q3: I mean +LSF-H2O2 vs -LSF+H2O2, they caused similar changes in the levels of C18:1 and C20:3, according to the Underlying Data. Why did these two treatments resulted in opposite biological outcomes, if C18:1 and C20:3 are key regulators?*

**

**Response:** We assume that the Reviewer means to compare LSF-H202 groups and LSF+H202 groups and not -LSF+H202 groups. There is no reason for us to compare +LSF-H2O2 vs -LSF+H2O2 groups as there are 2 variables in this comparison and it does not address our aim and hypothesis. Despite what the underlying data shows us (before statistical analysis is performed), I believe that Reviewer 2 would agree that it is common research practice to report the top fatty acids that are found to show differences in trends  (both significant p < 0.05 and non-significant p > 0.05) between the comparison groups as revealed by the MetaboAnalyst Software Version 3 but only discuss observations that are shown to be statistically significantly different. Hence, we have reported all top fatty acids as revealed by the analytical software to show the trends that may differ between the comparison groups regardless if they are significantly different or not but discussed only the observations that were validated by statistical analyses.

In our manuscript, we have shown that oleic acid (C18:1) and ETA (C20:3) were upregulated in LSF treatment groups compared to the untreated groups without oxidative stress stimulus.  Oleic acid is a monosaturated fatty acid (MUFA). We have discussed that the effects of LSF and oleic acid can be tissue-specific and that AMD patients demonstrated lower levels of oleic acid and omega-3 PUFAs in their red blood cells compared to their age-matched healthy controls (reference 32). In our Discussion, we also mentioned that an association study by Parekh et al (2009) demonstrated a correlation between a high MUFA diet and significantly reduced risk of AMD and this protective effect of MUFAs against AMD may involve anti-atherogenic pathways. The fact that LSF can increase MUFAs such as oleic acid may indicate its ability to compensate for low levels observed in AMD patients and possibly protect retinal cells against vascular anomalies seen in late AMD.

ETA is an omega-3 polyunsaturated fatty acid. In our Discussion, we have explained the the benefits of a high dietary intake of omega-3 and omega-9 fatty acids in alleviating the risk of AMD by about 30% to 40% and neovascularisation have been extensively reviewed by van Leeuwen *et al.* (2018). We also go on to explain that omega 3-PUFAs in the retina is said to be protective against photo-sensitised oxidation and peroxidation of lipids in the eyes of aging adults and hence may reduce expression of pro-angiogenic cytokines and subsequent choroid neovascularisation seen in advanced AMD (references 15 and 32). Therefore, LSF may be a naturally-occurring enhancer of omega-3 levels in RPE cells to protect RPE cells from inflammation and abnormal neovascularisation observed in AMD patients.

In view of the above, our observations and discussion points pertaining to oleic acid and ETA levels explain that these 2 fatty acids show the same trend upon LSF treatment and have similar biological effects in the context of maintaining retinal health and do not oppose.

As to why in the presence of H202 we do not observe the changes in these 2 fatty acids, this is not known as there are no other published literature that can explain this phenomenon. Perhaps, in the presence of stress, there are other compensatory mechanisms that may be involved in regulating anti-oxidative stress pathways (i.e. upregulating levels of GSTM1 enzymes) and hence related substrates such as selected fatty acids known to play a role in reversing oxidative stress. This has been discussed in our previous publication. Please see: https://www.ncbi.nlm.nih.gov/pubmed/29376497

*Q4: Based on your results, another 19 types of PEs are more abundant than 34:0 by the analyses of Prism. I quite understand that PE34:0 is not the LEAST abundant, as many others like 34:3 are even lower. In my analyses, the total PE level was significantly reduced in LSF20+H2O2, as compared with -LSF+H2O2 or LSF5+H2O2. This might be more related to your discussion on A2E production.*

**Response:** Indeed, our underlying data has revealed a variety of PE species that seem to be detected at lower levels in 20 µM LSF +H202 groups compared to the respective untreated controls. However, the Agilent Mass Hunter Quantitative Analysis software has revealed that only PE34:0 and PE38:5 are the two species that are found to be statistically different between the LSF and untreated groups in the presence of oxidative stress. Therefore, our discussion referred to PE34:0 and PE38:5 rather than other PE species. Furthermore, due to the novel nature of this study, there are no other reports up-to-date that discusses the probable relationship between LSF treatment of retinal cells, specific PE species and apolipoprotein. There are other studies such as https://www.ncbi.nlm.nih.gov/pubmed/?term=(Apolipoprotein%5BTitle%2FAbstract%5D)+AND+phosphatidylethanolamine+AND+Retina  that discusses PE levels in general (rather than specific PE species) and the role of apolipoproteins in primary retinal cells but this is under non-oxidative stress conditions and without LSF treatment. As a result, we discussed our observation regarding PE levels in a broader context of how PE lipids in general may be related to apolipoproteins in the retina and have included references to 44-46.  We can also only discuss what we observe (i.e. 20µM LSF + H202) with higher concentrations of LSF. As to why these PE species were not found to be statistically significant in the 5µM LSF +H202 groups, this may be due to a dose-dependent response. However, as there are no other papers that reveal similar information, this was not included in the discussion.

We hope that the above has addressed the Reviewer 2’s comments and kindly request Dr. Jacob Qi to approve our manuscript without any further reservations.

# Peer review: Reviewer response for version 2

**DOI:** 10.5256/f1000research.22941.r57430

**Published:** 2019-12-18

**Authors:** Yanfei (Jacob) Qi

**Affiliations:** 1 Centenary Institute, University of Sydney, Camperdown, NSW, Australia

LSF is a major antioxidant from cruciferous vegetables. Kwa *et al.* aim to validate if LSF protects human retinal epithelial cells from H2O2-induced oxidative damage and to identify the underlying molecular basis by employing lipidomic analyses. It may result in a better understanding of the pathogenesis of AMD. However, the following concerns should be better addressed.   

         

**Major concerns:**

1. In general, I don’t reckon that authors should split data interpretation into -/+ H2O2. It results in the loss of comparisons between – H2O2 and + H2O2 (in the absence of LSF) that is fundamental to demonstrate proper modelling of oxidative damage.
2. Fig. 1B. I cannot judge that effects of LSF result from protection against H2O2-induced cell death or an increased proliferation. 24 h treatment with H2O2 has been shown to cause toxicity in ARPE-19 cells ^1^ ^2^. The underlying raw data also demonstrate that the absorbance was reduced from 2.0+ to 1.0+ between CA-H2O2 (fig.1A) and CA+H2O2 (Fig.1b), if comparable. Did 2 h treatment with H2O2 cause any toxicity?  Following LSF pre-treatment, why the addition of H2O2 for only 2 h induced such dramatic proliferation? What is the doubling time of ARPE-19 cells? Please note, MTS assay measures the mitochondrial activity. To draw your conclusion of “increase in proliferation”, other assays should be carried out, e.g. BrdU incorporation and Ki67 staining. LSF may also result in the phenotype in Fig. 1b, via the regulation of the cell cycle ^3^.
3. Fig 2 and 3 show that in the absence of H2O2, LSF increased FAs C18:1 and C20:3. In the related discussion, authors believe that these increases contribute to LSF-mediated protection. However, as shown in the underlying data, the levels of C18:1 and C20:3 was higher or, at least, similar in H2O2-treated cells, as compared to LSF-treated samples. How to explain both LSF and H2O2 caused changes in the same way, whereas they were claimed to have opposite biological effects?
4. Fig. 4 and 5. In Fig. 5, authors need to double-check the fold change of PE 34:0, PE 38:5 and oPDA 34:3, which do not match the underlying data.  In addition, if you normalised the data over the untreated control, it is better to set the untreated control as 1, and show 20uM LSF in fold change. In the related discussions, 1) PE. Indeed, PE is the precursor for A2E production (ref 44). However, based on your lipidomics results, PE 34:0 is a less abundant PE species. Will the reduction of PE 34:0 “reduce the PE level” and thus determine the A2E production? Did you measure the A2E level? 2) PC. Similarly, the authors discussed the biofunction of overall PC in Bruch’s membrane. But the most abundant PC species in the retina are C 16:0/18:1, 18:0/18:1, 18:0/22:6, 18:2/20:4, etc ^4^. What is the physiological role of PC 33:3?

**Other concerns:**

1. In the figure legends, authors should clarify the treatment conditions, although the information has been mentioned somewhere else.
2. In fig. 4, authors should list lipid species following the order of lipid classification, but not in the current mixed pattern.
3. I suggest plotting the selected subspecies from all 6 experimental groups in Fig. 3 and 5. Then the ANOVA should be applied for multi-comparisons. T-tests on two groups are less critical.

Is the work clearly and accurately presented and does it cite the current literature?

Partly

If applicable, is the statistical analysis and its interpretation appropriate?

Partly

Are all the source data underlying the results available to ensure full reproducibility?

Yes

Is the study design appropriate and is the work technically sound?

Partly

Are the conclusions drawn adequately supported by the results?

Partly

Are sufficient details of methods and analysis provided to allow replication by others?

Yes

Reviewer Expertise:

Cell biology, lipid metabolism and lipidomics

I confirm that I have read this submission and believe that I have an appropriate level of expertise to confirm that it is of an acceptable scientific standard, however I have significant reservations, as outlined above.

## References

[1] : Role of quercetin in protecting ARPE‑19 cells against H2O2‑induced injury via nuclear factor erythroid 2 like 2 pathway activation and endoplasmic reticulum stress inhibition. *Mol Med Rep*.2017;16(3) : 10.3892/mmr.2017.6964 3461-3468 10.3892/mmr.2017.6964 28713895

[2] : Efficacy of Ethanol Extract of Fructus lycii and Its Constituents Lutein/Zeaxanthin in Protecting Retinal Pigment Epithelium Cells against Oxidative Stress: In Vivo and In Vitro Models of Age-Related Macular Degeneration. *J Ophthalmol*.2013;2013: 10.1155/2013/862806 862806 10.1155/2013/862806 24163760PMC3791792

[3] : Cell cycle effects of L-sulforaphane, a major antioxidant from cruciferous vegetables: The role of the anaphase promoting complex. *Hell J Nucl Med*.17 Suppl 1:11-6 24392461

[4] : Identification and quantification of phosphatidylcholines containing very-long-chain polyunsaturated fatty acid in bovine and human retina using liquid chromatography/tandem mass spectrometry. *J Chromatogr A*.2010;1217(49) : 10.1016/j.chroma.2010.10.039 7738-48 10.1016/j.chroma.2010.10.039 21035124

# Response

**Published:** 2020-02-13

**Authors:** Faith Kwa

**Affiliations:** RMIT University, Australia

Dear Reviewers 1 and 2, 

Thank you for the time you have taken to provide comments and suggestions. Please see our rebuttal to your comments below or via this document. 

**Rebuttal to Reviewer 1 Chris Barlow’s *second* set of comments. Please find our response below.**

*Upon reviewing this paper for a second time I’m afraid that I’m still unable to recommend it for indexing. The main results and conclusions from the abstract are not clearly supported by the data here. For example in the result section of the abstract the authors state: “LSF treatment also increased levels of the lipid classes phosphtidylcholine, cholesteryl ester and oxo-phytodienoic acid but decreased levels of phosphatidylethanolamine lipids.” This would suggest that the protection afforded by LSF treatment operates by altering the lipid profile prior to oxidative stress. Indeed, in the conclusion section of the introduction the authors propose that retinal cells can be pre-conditioned with LSF to regulate levels of selected fatty acids and lipids. However, from page 6 “In the absence of oxidative stress, no changes in lipid levels between LSF-treated cells and untreated control were reported.” So LSF pre-treatment does not alter the lipid profile although there are differences in the lipid profile following oxidative stress between cells that were LSF pre-treatment. These differences most likely reflect the reduction in oxidative damage in these cells rather than providing a rational for the mechanism of protection.*

**Response:** Our observations indicate that although under non-oxidative stress conditions, the addition of LSF to the cells did not result in significant changes to the lipid classes stated by Reviewer 1, there were significant changes in these lipid classes when the cells were pre-treated with LSF prior to the exposure of hydrogen-peroxide when compared with untreated cells under the same stress conditions. These observations suggest that LSF may only result in very subtle but undetectable changes in lipids which may be masked minimal changes in the level of enzymes required for lipid metabolism and transport ( https://www.sciencedirect.com/science/article/pii/S0005273617301220). However, when cells are pre-treated with LSF and subsequently exposed to oxidative stress, the activation of genes encoding such enzymes may be triggered in the presence of stress and disease ( https://www.mdpi.com/1420-3049/24/18/3231/htm) which may account for the said changes in the lipid levels. This phenomenon may be due to the fact that LSF is a known histone deacetylase inhibitor ( https://www.sciencedirect.com/science/article/pii/S1359644614001202?via%3Dihub), it may induce an epigenetic memory in cells without causing any changes in gene expression under normal circumstances but actives genes when necessary (i.e. under cellular stress). Therefore, we have proposed investigations surrounding the effects of LSF on expression of genes that regulate lipid metabolism and transport to form part of our future studies as already mentioned in our manuscript. Despite this our observations still supports the conclusion made that cells can be pre-conditioned with LSF to prepare them for subsequent oxidative stress and assist their recovery should cellular insult occurs (as evidenced by our cell proliferation data). We have also now amended the final sentence in the manuscript to read “These further studies will facilitate the design of targeted therapies that can be co-administered with LSF for the management of AMD progression”.

*There also appear to be discrepancies between the authors response to my previous review and the changes made in the revised version of the manuscript. For example, in their response to my previous review:*

*We have amended the Methods section by removing the use of calibration curves and added the statement “The data was generated using the reverse phase peak area response of each lipid species rather than the absolute concentration.”*

*I was unable to find this statement in the revised version of the manuscript, instead on page 5 we have: “The lipidomic data was generated using the peak ion area response of each lipid series relative to an internal standard rather than absolute concentrations using reverse phase LC-MS”.*

**R** **esponse:** The amended statement appears to be omitted during the processing and publication of the revised version of the manuscript. This statement has now been included in Version 3 of the manuscript under the subheading “Lipid analysis under LC-MS” in the Methods section.

*So, did the authors use internal standards or not? d7-cholesterol is the only lipidomics standard that I could find mentioned in the text although it doesn’t seem to be included in the repository data. The repository data does include internal standards for LPC, PI, SM and TG.*

**Response:** To clarify, the raw data was first normalised to d7-cholesterol using the Mass Hunter Quant V6 software and then exported into Excel for further normalisation by manual calculation using cell numbers per sample (formula: median reverse phase peak area under the chromatogram divide by cell number). The normalised data is then exported into MetaboAnalyst for further analysis and generation of heatmaps and box-whisker plots. However, the raw data presented exported using Mass Hunter will not show the d7-cholesterol peak area and hence not presented. We have removed the section in the Methods which refers to normalisation to d7-cholesterol to avoid confusion.

To reiterate, the original lipid data was further normalised to cell numbers and then median, log-transformed and autoscaled to generate results (eg heatmap). Here is an example showing lipid data not normalised to cell numbers. It can clearly be seen that even after applying post-normalisation parameters (i.e. median, log-transform and autoscaled) the lipid data is normally distributed. Please note that the graphs shown list only a portion lipids of a non-exhaustive list and therefore the lipids you see here may not necessarily show the same lipids in the heatmap data within the manuscript which shows the top 25 lipids where differences are found between the untreated and 20µM LSF groups.

Furthermore, with the normalisation to both cell number and median reverse phase peak area, there are no changes in the significant lipid species before or after normalisation as shown in an example of data analysis below. Normalising the data to cell number is appropriate, as the cell number varies between replicates, before plotting the data using Metaboanalyst. We have not included these heatmaps here because the heatmap in the manuscript is identical before and after normalisation.

*In a similar vein, my previous review made mention of several fold-changes that were in error which the authors attributed to typographical errors. The authors claim to have amended these values in the revised version of the manuscript. In the revised version of the manuscript however we find that all of the fold-changes have been altered, including those which were previously in agreement with the reviewer’s calculation. In the revised submission all values are now in disagreement with both the original submission and this reviewers’ calculations (see here). https://f1000researchdata.s3.amazonaws.com/linked/269780.Chris_Barlow_v2_table_for_IM.JPG*

**Response:** We respectfully request Reviewer 1 to provide the calculation steps. We have used the open-source MetaboAnalyst Version 3 and performed the statistical analysis using the Bonferroni-adjusted values to analyse the fold-changes reported. Despite difference in the Reviewer’s calculations, the trends of lipid changes remain the same which still align with the discussion and conclusions made.

*Some aspects of the data analysis continue to be unclear. For example, the lipidomic data was normalized based on the number of cells (these numbers should be provided in the repository for clarity) which seems extremely sensible. However, the authors state that the data was also normalized to the median of the peak area response. As the median peak area response would also scale with the number of cells wouldn’t this second normalization negate the normalization by the number of cells? Why is it necessary to normalize to the median value? Finally, the authors state that “An autoscale has also been applied.” What is the auto-scale and how has it been applied?*

**Response:** We have already provided Reference 27 to support the use of  an auto-scale which is routinely used by the Metabolomics community to visualise data. According to MetaboAnalyst, the auto-scale is equal to: mean-centered and divided by the standard deviation of each variable.

As there were differences between the cell numbers across each set of replicates, we normalised the data against the cell number and then against the median peak area response to limit the influence of the variable cell numbers on the data analysis.

Cell numbers are now provided in the underlying data Version 3.

*The authors state in the statistical and data analysis section that a paired t-test was used for the lipidomic analysis. Given the study design it is not clear to me the basis on which the samples were “paired”.*

*The authors also need to adjust their analysis to account for the multiple comparisons problem. This was raised in my previous review but the authors appear to have misunderstood my meaning, discussing instead comparisons of multiple groups by ANOVA analysis.*

**Response: **To address this concern, we have now amended the statement and specified under the subheading ‘Statistical and data analyses’: “Significant changes in cell proliferation and levels of total fatty acids or lipid species were validated by one-way analysis of variance and the post-hoc Bonferroni test and paired t-test to determine any significant differences between the treated groups (5µM, 10µM, 20µM and/or 30µM LSF) and untreated or vehicle controls”. This analysis was performed by MetaboAnalyst Version 3 as mentioned in the manuscript.

*To clarify I’m talking about the problem of false positive identifications that will arise when comparing a large number of analytes. In the current example the authors consider 328 lipids, with a 5% cut-off we expect 16.4 lipids to have a p-value of <0.05 by chance alone. To combat this problem people typically employ something like a Bonferroni correction (which the authors have apparently used in their fatty acid analysis) or more often the Benjamini-Hochberg procedure. The authors need to demonstrate that the lipids reported as significantly different remain statistically significant upon application of this more stringent test. I would also recommend reporting the p-values here.*

**Response:** The data analyses has already incorporated the Bonferroni correction. The p-values which derive from the t-test and ANOVA are now included in Version 3 of the manuscript.

*I have some additional comments:*

*In Figure 3 plotting the points from the untreated control is misleading as all of these points have been imputed.*

**Response: **We have addressed this point in our earlier rebuttal. We have removed any mention of fold changes in fatty acid levels in the manuscript and in Figure 3, but indicated that a comparison in the detection levels between untreated and treated groups. We have also removed any references to p values when reporting fatty acid data. Figure 3’s aim is to depict the trends in different levels of fatty acids between the treated and untreated groups.

*In Figure 5 I note that PC 33:3 is plotted twice and somewhat surprisingly the plots do not match.*

**Response:** We thank Reviewer 1 for detecting this anomaly. PC33:3.1 was a replicate in the set of 20µM LSF + treatment group. Subtle differences between the replicate and the averaged data point may not be apparent in the heatmap. The data for this replicate has been now been removed and Figures 4B and 5 have been updated.

**Rebuttal to Reviewer 2 Jacob Qi’s comments. Please find our response below.**

*LSF is a major antioxidant from cruciferous vegetables. Kwa et al. aim to validate if LSF protects human retinal epithelial cells from H2O2-induced oxidative damage and to identify the underlying molecular basis by employing lipidomic analyses. It may result in a better understanding of the pathogenesis of AMD. However, the following concerns should be better addressed.*

*Major concerns: 1.In general, I don’t reckon that authors should split data interpretation into -/+ H2O2. It results in the loss of comparisons between – H2O2 and + H2O2 (in the absence of LSF) that is fundamental to demonstrate proper modelling of oxidative damage.*

**Response:** The in vitro oxidative stress model we have used is widely validated in the ARPE-19 cell line ( https://ekjo.org/DOIx.php?id=10.3341/kjo.2003.17.1.19 and https://www.ncbi.nlm.nih.gov/pubmed/29376497) . The purpose of splitting the data into -/+ H2O2 are to assess the net effects of LSF on the cells under normal conditions and oxidative stress conditions. It is important to establish that LSF does not exert any toxicity on the cells under normal circumstances and if it was toxic, this would challenge our hypothesis that LSF can be used as a supplement in people at risk of AMD or have early stages of the disease where it is characterised by oxidative damage in retinal cells. Therefore, for the consistency of data presentation throughout the manuscript, all data reported have been presented in this format.

*2.Fig. 1B. I cannot judge that effects of LSF result from protection against H2O2-induced cell death or an increased proliferation. 24 h treatment with H2O2 has been shown to cause toxicity in ARPE-19 cells 12. The underlying raw data also demonstrate that the absorbance was reduced from 2.0+ to 1.0+ between CA-H2O2 (fig.1A) and CA+H2O2 (Fig.1b), if comparable. Did 2 h treatment with H2O2 cause any toxicity?  Following LSF pre-treatment, why the addition of H2O2 for only 2 h induced such dramatic proliferation? What is the doubling time of ARPE-19 cells? Please note, MTS assay measures the mitochondrial activity. To draw your conclusion of “increase in proliferation”, other assays should be carried out, e.g. BrdU incorporation and Ki67 staining. LSF may also result in the phenotype in Fig. 1b, via the regulation of the cell cycle 3.* 

**Response: **As the study outlined in this manuscript is an extension of our first paper published in 2018 ( https://www.ncbi.nlm.nih.gov/pubmed/29376497), we have already reported the toxic effects of 200µM H2O2 in Figure 2 of this previous paper. In addition, other studies have also used H2O2 concentrations of 100µM to low mM concentrations in oxidative stress cell-based models ( https://iopscience.iop.org/article/10.1088/1752-7163/ab1fc4/pdf). As referenced in our earlier paper, the ARPE-10 cells have been starved in a serum-deprived DMEM/F12

media containing 1% FCS and 1% penicillin-streptomycin for 24 hours so that all the cells are synchronized at the same cell cycle phase and to remove time as a factor for cell proliferation. This is common practice in *in vitro* assays ( https://journals.plos.org/plosone/article/file?id=10.1371/journal.pone.0120587&type=printable, https://www.ncbi.nlm.nih.gov/pmc/articles/PMC3329488/ and file://rmit.internal/USRHome/eh2/E02862/Downloads/z7g00906004098.pdf). The MTS assay used in this study is a commercially available Cell Proliferation Assay from Promega and has been widely used to determine proliferation and cytotoxicity based on the measurement of lactate dehydrogenase enzymes in the metabolically active cells. Further references are provided in the manufacturer’s protocol ( https://www.promega.com/-/media/files/resources/protocols/technical-bulletins/0/celltiter-96-aqueous-one-solution-cell-proliferation-assay-system-protocol.pdf).

*3.Fig 2 and 3 show that in the absence of H2O2, LSF increased FAs C18:1 and C20:3. In the related discussion, authors believe that these increases contribute to LSF-mediated protection. However, as shown in the underlying data, the levels of C18:1 and C20:3 was higher or, at least, similar in H2O2-treated cells, as compared to LSF-treated samples. How to explain both LSF and H2O2 caused changes in the same way, whereas they were claimed to have opposite biological effects?* 

**Response:** Figures 2 and 3 show the trend differences in fatty acid levels between LSF treated cells and the untreated controls in the absence of oxidative stress. The underlying data shows that the raw values of the replicates between LSF treated cells and untreated controls in the presence of oxidative stress are relatively similar and may account for any lack in significant trend differences in the “+H2O2” groups. I compared the underlying values for LSF-H202 groups and LSF+H202 groups. Although it appears that values of the LSF-treated cells in the “+H2O2” groups are slightly higher to that of those in the “-H2O2” groups, there is some variation in cell numbers (version 3 of data in repository) across the replicates within each of these “+H2O2” groups.  The “increased values” could possibly be due to the effects of hydrogen peroxide since the variable between the two groups is the presence of H2O2 but without rigorous statistical analysis, this conclusion cannot be made. It is important to note that the aim of Figures 2 and 3 is to show if the variable in this case, LSF treatment, changes the levels of the fatty acids in comparison to the untreated control and this is indeed what we observed which supports our hypothesis that LSF treatment can precondition cells to adapt to subsequent oxidative stress which may involve regulating levels of fatty acids that are known to play an important role in oxidative damage recovery. The direct relationship of how these fatty acids regulate the protective effects of LSF needs to be investigated in future studies as outlined in the manuscript.

*4.Fig. 4 and 5. In Fig. 5, authors need to double-check the fold change of PE 34:0, PE 38:5 and oPDA 34:3, which do not match the underlying data.  In addition, if you normalised the data over the untreated control, it is better to set the untreated control as 1, and show 20uM LSF in fold change.* 

**Response: **Kindly note that the underlying data depicts the raw values generated by the mass spectrophotometer before any data analysis is performed where the figures show data normalised to cell numbers and then to median peak area. The heatmaps and boxplots were automatically generated by MetaboAnalyst Software Version 3 using the normalised data. Thus, the values shown in the figures do not match that of the underlying data. The fold changes were determined by: Median peak area of the lipid in the treatment group/median peak area of the lipid in the respective control group.

*In the related discussions, 1) PE. Indeed, PE is the precursor for A2E production (ref 44). However, based on your lipidomics results, PE 34:0 is a less abundant PE species. Will the reduction of PE 34:0 “reduce the PE level” and thus determine the A2E production? Did you measure the A2E level? 2) PC. Similarly, the authors discussed the biofunction of overall PC in Bruch’s membrane. But the most abundant PC species in the retina are C 16:0/18:1, 18:0/18:1, 18:0/22:6, 18:2/20:4, etc 4. What is the physiological role of PC 33:3?*

**Response:** We would like to respectfully ask how Reviewer 2 came to a conclusion that PE34:0 is a less abundant PE species and in comparison to which other PE species? The underlying data indicate the values of some PE species such as PE34:3 are even lower than that of PE34:0. We have not measured the levels of A2E in this study this can be included as a future study to validate if reducing PE34:0 and PE38:5 can reduce A2E levels. We have now included this as a future study in the revised manuscript. Unfortunately, the relationship between PC33:3 and retinal function or AMD is not well established and hence we discussed the general biofunction of PCs in the Bruch’s membrane. However, according to the Metabolomics Innovation Centre website, PC33:3 which can be made up of (15:0/18:3) and is said to be involved in phospholipid metabolism, lipid transport, lipid metabolism, lipid peroxidation etc. Please see here: http://www.hmdb.ca/metabolites/HMDB0007941 .

*Other concerns: 1.In the figure legends, authors should clarify the treatment conditions, although the information has been mentioned somewhere else.*

**Response: **Although the treatment conditions are often found in the figure title, the figure legends have now been expanded to include information on the treatment conditions, where previous information is vague.

*2.In fig. 4, authors should list lipid species following the order of lipid classification, but not in the current mixed pattern.* 

**Response: **As mentioned above, the heatmaps were automatically generated by MetaboAnalyst Software Version 3 using the analysed data. We are not able to change the way this data is presented.

*3.I suggest plotting the selected subspecies from all 6 experimental groups in Fig. 3 and 5. Then the ANOVA should be applied for multi-comparisons. T-tests on two groups are less critical.* 

**Response:**The boxplots were automatically generated by MetaboAnalyst Software Version 3 using the analysed data. However, boxplots were only generated by the software where statistically significant changes between the treated and untreated groups were detected. Reference 27 which has been provided in the manuscript supports the routine use of MetaboAnalysis Software Version 3 in analysing metabolomic-based data. Pairwise T test compares two groups (i.e. treatment group versus untreated group) while the ANOVA was performed for multiple comparison of all groups. Both types of statistical analyses have been done in the study. The p-values generated by the ANOVA analysis have now been included in the respective Results section of the revised manuscript.

# Peer review: Reviewer response for version 2

**DOI:** 10.5256/f1000research.22941.r55259

**Published:** 2019-11-19

**Authors:** Chris Barlow

**Affiliations:** 1 Monash University, Melbourne, Vic, Australia

Upon reviewing this paper for a second time I’m afraid that I’m still unable to recommend it for indexing. The main results and conclusions from the abstract are not clearly supported by the data here. For example in the result section of the abstract the authors state: “LSF treatment also increased levels of the lipid classes phosphtidylcholine, cholesteryl ester and oxo-phytodienoic acid but decreased levels of phosphatidylethanolamine lipids.” This would suggest that the protection afforded by LSF treatment operates by altering the lipid profile prior to oxidative stress. Indeed, in the conclusion section of the introduction the authors propose that retinal cells can be pre-conditioned with LSF to regulate levels of selected fatty acids and lipids. However, from page 6 “In the absence of oxidative stress, no changes in lipid levels between LSF-treated cells and untreated control were reported.” So LSF pre-treatment does not alter the lipid profile although there are differences in the lipid profile following oxidative stress between cells that were LSF pre-treatment. These differences most likely reflect the reduction in oxidative damage in these cells rather than providing a rational for the mechanism of protection.

 There also appear to be discrepancies between the authors response to my previous review and the changes made in the revised version of the manuscript. For example, in their response to my previous review:

 We have amended the Methods section by removing the use of calibration curves and added the statement “The data was generated using the reverse phase peak area response of each lipid species rather than the absolute concentration.”

 I was unable to find this statement in the revised version of the manuscript, instead on page 5 we have: “The lipidomic data was generated using the peak ion area response of each lipid series relative to an internal standard rather than absolute concentrations using reverse phase LC-MS”.

 So, did the authors use internal standards or not? d7-cholesterol is the only lipidomics standard that I could find mentioned in the text although it doesn’t seem to be included in the repository data. The repository data does include internal standards for LPC, PI, SM and TG.

 In a similar vein, my previous review made mention of several fold-changes that were in error which the authors attributed to typographical errors. The authors claim to have amended these values in the revised version of the manuscript. In the revised version of the manuscript however we find that all of the fold-changes have been altered, including those which were previously in agreement with the reviewer’s calculation. In the revised submission all values are now in disagreement with both the original submission and this reviewers’ calculations (see here).

 Some aspects of the data analysis continue to be unclear. For example, the lipidomic data was normalized based on the number of cells (these numbers should be provided in the repository for clarity) which seems extremely sensible. However, the authors state that the data was also normalized to the median of the peak area response. As the median peak area response would also scale with the number of cells wouldn’t this second normalization negate the normalization by the number of cells? Why is it necessary to normalize to the median value? Finally, the authors state that “An autoscale has also been applied.” What is the auto-scale and how has it been applied?

 The authors state in the statistical and data analysis section that a paired t-test was used for the lipidomic analysis. Given the study design it is not clear to me the basis on which the samples were “paired”.

 The authors also need to adjust their analysis to account for the multiple comparisons problem. This was raised in my previous review but the authors appear to have misunderstood my meaning, discussing instead comparisons of multiple groups by ANOVA analysis.

 To clarify I’m talking about the problem of false positive identifications that will arise when comparing a large number of analytes. In the current example the authors consider 328 lipids, with a 5% cut-off we expect 16.4 lipids to have a p-value of <0.05 by chance alone. To combat this problem people typically employ something like a Bonferroni correction (which the authors have apparently used in their fatty acid analysis) or more often the Benjamini-Hochberg procedure. The authors need to demonstrate that the lipids reported as significantly different remain statistically significant upon application of this more stringent test. I would also recommend reporting the p-values here.

 I have some additional comments:

 In Figure 3 plotting the points from the untreated control is misleading as all of these points have been imputed.

 In Figure 5 I note that PC 33:3 is plotted twice and somewhat surprisingly the plots do not match.

Is the work clearly and accurately presented and does it cite the current literature?

Partly

If applicable, is the statistical analysis and its interpretation appropriate?

No

Are all the source data underlying the results available to ensure full reproducibility?

Yes

Is the study design appropriate and is the work technically sound?

Partly

Are the conclusions drawn adequately supported by the results?

No

Are sufficient details of methods and analysis provided to allow replication by others?

Partly

Reviewer Expertise:

NA

I confirm that I have read this submission and believe that I have an appropriate level of expertise to state that I do not consider it to be of an acceptable scientific standard, for reasons outlined above.

# Peer review: Reviewer response for version 1

**DOI:** 10.5256/f1000research.21489.r52230

**Published:** 2019-08-08

**Authors:** Chris Barlow

**Affiliations:** 1 Monash University, Melbourne, Vic, Australia

**Overview:**

 This paper seeks to build on previous work published by this group which has demonstrated that pre-treatment of ARPE-19 cells with L-Sulforaphane (LSF) leads to protection against oxidative damage as assessed using a cell proliferation assay. In particular, here the authors seek to identify any changes in fatty acids and more complex lipids following pre-treatment with LSF and then H _2_O _2_. The authors report that no fatty acids (total fatty acid analysis) were significantly altered under conditions of oxidative stress. However, they report extremely large fold-changes for *cis*-oleic, *trans*-oleic and eicosatrienoic acids upon treatment with LSF in the absence of oxidative stress. For the lipidomic portion of the study the authors report that several lipids showed statistically significant differences under oxidative stress following pre-treatment with LSF. Unfortunately, the paper suffers from problems associated with data analysis making it unsuitable for indexing at this time.

**Fatty acid analysis:**

 On page 5 the authors report very large fold-changes on the order of 10 ^7^ to 10 ^9^ for oleic ( *cis* and *trans*) and ETA upon treatment with LSF in the absence of oxidative stress. Upon inspection of the underlying data I found that for all four of the "Untreated –" samples, the intensity for these fatty acids was 1x10 ^-7^ for both *cis*- and *trans*- oleic acid and 1x10 ^-9^ for ETA (see table here). It would appear that these fatty acids were not detected in these samples and subsequently values have been imputed automatically. While imputation is common in metabolomic analysis it is not valid to report a fold-change or p-value where all values for the control group have been imputed.

**Lipidomic Analysis:**

 Here the authors report that several lipids were significantly different (p < 0.001) upon pre-treatment with 20 µM LSF relative to the untreated controls under conditions of oxidative stress. Careful examination of the underlying data suggests that a couple of errors may have been made in data processing. Specifically, the fold-change for PC 33:3 was 1.946 and not 2.33 as reported. Similarly, PE 34:0 and PE 38:5 had fold-changes of 0.723 and 0.684 respectively and not the reported 0.394 and 0.292 reported in the paper. I have reproduced the relevant data from the underlying data in the table here for clarity. I’m also confused about the statistical analysis. Using a two-tailed t-test none of the lipids reported as significant had a p-value < 0.001 as reported in the text, more generally it is unclear how the authors have dealt with the issue of multiple comparisons. The experimental section of lists several statistical tests but it is unclear which test was used for each analysis. Greater clarification as to how the statistical analysis was performed is necessary.

**Experimental Design:**

 The authors have used n = 4 for each group. While n = 4 is probably fine for using assays with a single metric such as the proliferation assay it is probably insufficient for lipidomic analysis. Indeed, the lipid data seems to be highly variable. For example, the total amount of lipid seems to vary substantially more than I would have expected. A crude measure of this variation is reflected in the median intensity of all the lipid measured for each sample which I have plotted here.

 From the experimental section on page 4 it appears that cells were seeded at a density of 1.5 x 10 ^6^ before being conditioned as described in the “Cell treatment prior to analysis” section. If I’m reading this correctly then there was no adjustment for the number of cells following the conditioning but immediately before lipid extraction. Were an equivalent number of cells extracted (as opposed to seeded) in each sample, and if so, do the authors have any insight into why such a high degree of variability was observed in the lipidomics data? Similarly, I assume that treatment with hydrogen peroxide resulted in some cell death, what steps were taken to ensure that dead cells were not being extracted along with the live cells?

 Finally, I would suggest that some important comparisons have not been analysed. Presumably the hypothesis is that LSF treatment leads to changes in lipid profile which are then somehow protective against oxidative damage. I would suggest that the authors need to compare the lipid profiles of the LSF 20 µM – against the untreated – group. This should capture lipidomic differences associated with LSF treatment without the confounding effect of H _2_O _2_ treatment. 

**Additional notes and suggestions:**

- On page 3 the authors state that all five major fatty acids in the human retina are long chain polyunsaturated fatty acids (PUFAs). This is incorrect, docosahexaenoic and arachidonic acids are PUFAs, oleic acid is monounsaturated and stearic and palmitic are saturated.
- Figures 2 and 4: The annotations are too small to read. The graphs need to be re-drawn with a larger font.
- Figure 3 and 5: The authors state that the y-axis values are automatically generated as arbitrary units by the software used. The authors need to clearly state how the data is being processed. (In this case it seems likely that the data is being log _2_ transformed.) Is a box and whisker plot appropriate for four data points?
- Page 5: The text here indicates that lipid quantitation was performed using external calibration curves however no concentrations are given throughout the text or underlying data.
- In the “LCMS Lipid Analysis Data.xlsx” file from the underlying data, five lipids are included in duplicate; PC 31:1, PC 33:3, PC 33:2, PC 34:4 and PC 37:4.

**Conclusion:**

 Due to the problems with the data analysis outlined above I’m unable to recommend this paper for indexing. If the authors are able to address these problems however, this paper should be considered as a fresh submission.

Is the work clearly and accurately presented and does it cite the current literature?

Partly

If applicable, is the statistical analysis and its interpretation appropriate?

No

Are all the source data underlying the results available to ensure full reproducibility?

Yes

Is the study design appropriate and is the work technically sound?

Partly

Are the conclusions drawn adequately supported by the results?

No

Are sufficient details of methods and analysis provided to allow replication by others?

Partly

Reviewer Expertise:

NA

I confirm that I have read this submission and believe that I have an appropriate level of expertise to state that I do not consider it to be of an acceptable scientific standard, for reasons outlined above.

# Response

**Published:** 2019-10-04

**Authors:** Faith Kwa

**Affiliations:** RMIT University, Australia

**Rebuttal to Reviewer 1 Chris Barlow’s comments. Please find our response below.**

**Overview:**

This paper seeks to build on previous work published by this group which has demonstrated that pre-treatment of ARPE-19 cells with L-Sulforaphane (LSF) leads to protection against oxidative damage as assessed using a cell proliferation assay. In particular, here the authors seek to identify any changes in fatty acids and more complex lipids following pre-treatment with LSF and then H2O2. The authors report that no fatty acids (total fatty acid analysis) were significantly altered under conditions of oxidative stress. However, they report extremely large fold-changes for cis-oleic, trans-oleic and eicosatrienoic acids upon treatment with LSF in the absence of oxidative stress. For the lipidomic portion of the study the authors report that several lipids showed statistically significant differences under oxidative stress following pre-treatment with LSF. Unfortunately, the paper suffers from problems associated with data analysis making it unsuitable for indexing at this time.

**Response:** We thank Reviewer 1’s concise summary of the work presented in our manuscript. However, we have to respectfully disagree that the data analysis using one-way ANOVA and paired t-test is not appropriate for the nature of the work here.  The data was always analysed at the significance level of p<0.05 but where p values generated were indeed less than 0.001, we have indicated this as p<0.001. We have now clearly indicated a statistically significant level of p<0.05 in the Methods section. This significance level and both statistical tests used in the fatty acid and lipid data here are standard statistical packages recommended by MetaboAnalyst Version 2 which we have already referenced in Reference 27. For the fatty acids where fold-changes for cis-oleic, trans-oleic and eicosatrienoic acids upon treatment with LSF in the absence of oxidative stress were reported, the untreated groups had an amount of fatty acids that were below the level of detection and hence for the purpose of performing univariate statistics, the missing values were imputed in alignment with common practice in metabolomics analysis as acknowledged by the Reviewer below. Taking the reviewer’s comments into consideration, we have edited the Result section to indicate that the levels of these fatty acids were only detected in the respective LSF treatment groups but not in the untreated controls. We have also removed any mention of fold changes in fatty acid levels in the manuscript and in Figure 3 but indicated that a comparison in the detection levels between untreated and treated groups. We have also removed any references to p values when reporting fatty acid data.

On page 5 the authors report very large fold-changes on the order of 107 to 109 for oleic (cis and trans) and ETA upon treatment with LSF in the absence of oxidative stress. Upon inspection of the underlying data I found that for all four of the "Untreated –" samples, the intensity for these fatty acids was 1x10-7 for both cis- and trans- oleic acid and 1x10-9 for ETA (see table here). It would appear that these fatty acids were not detected in these samples and subsequently values have been imputed automatically. While imputation is common in metabolomic analysis it is not valid to report a fold-change or p-value where all values for the control group have been imputed.

**Response:** Kindly see our response to the previous comment.

**Lipidomic Analysis:**

Here the authors report that several lipids were significantly different (p < 0.001) upon pre-treatment with 20 µM LSF relative to the untreated controls under conditions of oxidative stress. Careful examination of the underlying data suggests that a couple of errors may have been made in data processing. Specifically, the fold-change for PC 33:3 was 1.946 and not 2.33 as reported. Similarly, PE 34:0 and PE 38:5 had fold-changes of 0.723 and 0.684 respectively and not the reported 0.394 and 0.292 reported in the paper. I have reproduced the relevant data from the underlying data in the table here for clarity. I’m also confused about the statistical analysis. Using a two-tailed t-test none of the lipids reported as significant had a p-value < 0.001 as reported in the text, more generally it is unclear how the authors have dealt with the issue of multiple comparisons. The experimental section of lists several statistical tests but it is unclear which test was used for each analysis. Greater clarification as to how the statistical analysis was performed is necessary.

**Response:** We thank Reviewer 1 for pointing out the typographical errors which have been amended in the revised version of the manuscript. Regarding the statistical analyses, we used one-way ANOVA to analyse the GCMS and LCMS data presented in the heat maps that illustrates how the expression of fatty acids or lipids differ with each treatment or oxidative stress/normal conditions. Therefore, a multiple comparison was made determine the effect of LSF treatment and oxidative stress across the various groups. In contrast, a paired t test was used to analyse the GCMS and LCMS data represented in the box plots. This was a direct comparison between the fold change levels seen in the untreated control and those in the groups treated with 20 µM LSF. We did not use the two-tailed t-test. We would also like to clarify that we used the statistical significance level of p<0.05 and not p <0.001 in MetaboAnalyst Version 2. The revised manuscript has been corrected to indicate where p values are less than 0.05 instead of 0.001. 

**Experimental Design:**

The authors have used n = 4 for each group. While n = 4 is probably fine for using assays with a single metric such as the proliferation assay it is probably insufficient for lipidomic analysis. Indeed, the lipid data seems to be highly variable. For example, the total amount of lipid seems to vary substantially more than I would have expected. A crude measure of this variation is reflected in the median intensity of all the lipid measured for each sample which I have plotted here.

**Response:**Due to the complexity of the study, it is challenging to generate a higher number of replicates for this study. A minimum of 1.5 million cells were seeded for each of the four replicates per control and treatment groups (i.e. a minimum of 36 million cells used in the metabolomics investigations). There are many papers reporting lipidomic/ LCMS studies which analyse data from less than 4 repeat experiments. These include recent articles published in Q1 journals such as Oncology Reports and PLOS Biology. Examples can be found in the links below:

https://www.spandidos-publications.com/10.3892/or.2018.6510

https://journals.plos.org/plosbiology/article?id=10.1371/journal.pbio.2002214

From the experimental section on page 4 it appears that cells were seeded at a density of 1.5 x 106 before being conditioned as described in the “Cell treatment prior to analysis” section. If I’m reading this correctly then there was no adjustment for the number of cells following the conditioning but immediately before lipid extraction. Were an equivalent number of cells extracted (as opposed to seeded) in each sample, and if so, do the authors have any insight into why such a high degree of variability was observed in the lipidomics data? Similarly, I assume that treatment with hydrogen peroxide resulted in some cell death, what steps were taken to ensure that dead cells were not being extracted along with the live cells?

**Response:**  Although not apparent in our Methods section of the manuscript, during cell harvesting prior to lipid extraction, we washed the wells containing the adherent cells with PBS (pH 7.4) to remove any dead cells and cell debris. Following trypsinisation of the adherent cells per well, we performed a live cell count using the trypan blue exclusion method. Furthermore, the data has been normalised to both the number of cells per sample and the median of the reverse phase peak area response using the MetaboAnalyst Software Version 2 which will reduce any impact of variation by any differences in cell numbers. We have added these details in the revised version of the manuscript.

Finally, I would suggest that some important comparisons have not been analysed. Presumably the hypothesis is that LSF treatment leads to changes in lipid profile which are then somehow protective against oxidative damage. I would suggest that the authors need to compare the lipid profiles of the LSF 20 µM – against the untreated – group. This should capture lipidomic differences associated with LSF treatment without the confounding effect of H2O2 treatment.

**Response:** We thank Reviewer 1 for his recommendation. We have indeed compared fatty acid and lipid levels between LSF 20 µM and untreated groups in the presence and absence of H _2_O _2_. This is already stated in the manuscript in the first line of the relevant paragraph: “In the absence of oxidative stress, no changes in lipid levels between LSF-treated cells and the untreated control were reported (all *p* values > 0.05; see *Underlying data*)”. However, we only had presented figures where significant differences were established using the statistical tests described.

Additional notes and suggestions:•On page 3 the authors state that all five major fatty acids in the human retina are long chain polyunsaturated fatty acids (PUFAs). This is incorrect, docosahexaenoic and arachidonic acids are PUFAs, oleic acid is monounsaturated and stearic and palmitic are saturated.

**Response:** We have amended this statement to “There are five major fatty acids in the human retina, namely, docosahexaenoic acid (DHA), arachidonic acid (ACA), stearic acid, oleic acid and palmitic acid. Both DHA and ACA are classified as long chain polyunsaturated fatty acids (LC-PUFAs).”

•Figures 2 and 4: The annotations are too small to read. The graphs need to be re-drawn with a larger font.

**Response:**We have enlarged the font in these figures in the revised version of the manuscript.

•Figure 3 and 5: The authors state that the y-axis values are automatically generated as arbitrary units by the software used. The authors need to clearly state how the data is being processed. (In this case it seems likely that the data is being log2 transformed.) Is a box and whisker plot appropriate for four data points?

**Response:**All the data has been normalised to the median of the reverse phase peak area response and log2 transformed and number of cells per sample. An auto-scale has also been applied. These statements have been added to the Methods section of the revised manuscript. A box and whisker plot is one of the standard ways to present data generated by Metaboanalyst 2.0.

•Page 5: The text here indicates that lipid quantitation was performed using external calibration curves however no concentrations are given throughout the text or underlying data.

**Response:** All the figures were generated using reverse phase peak area response of each lipid species rather than the absolute concentration. We did not have standards for some of the lipid classes and therefore, to be consistent, we have used such responses to make comparison between the untreated and treated groups. We have amended the Methods section by removing the use of calibration curves and added the statement “The data was generated using the reverse phase peak area response of each lipid species rather than the absolute concentrations”.

•In the “LCMS Lipid Analysis Data.xlsx” file from the underlying data, five lipids are included in duplicate; PC 31:1, PC 33:3, PC 33:2, PC 34:4 and PC 37:4.

**Response:** We have removed the duplicated columns and reuploaded the underlying data onto the Version 2 of the Harvard Dataverse link.

Conclusion:

Due to the problems with the data analysis outlined above I’m unable to recommend this paper for indexing. If the authors are able to address these problems however, this paper should be considered as a fresh submission.

****

**Response:** We believe that our revised manuscript following the inclusion of the recommended edits deserves another peer-review and approval for publication in F1000 Research. The data has been statistically validated and does support the conclusions made in this manuscript.
